# A novel druggable interprotomer pocket in the capsid of rhino- and enteroviruses

**DOI:** 10.1371/journal.pbio.3000281

**Published:** 2019-06-11

**Authors:** Rana Abdelnabi, James A. Geraets, Yipeng Ma, Carmen Mirabelli, Justin W. Flatt, Aušra Domanska, Leen Delang, Dirk Jochmans, Timiri Ajay Kumar, Venkatesan Jayaprakash, Barij Nayan Sinha, Pieter Leyssen, Sarah J. Butcher, Johan Neyts

**Affiliations:** 1 Department of Microbiology and Immunology, Rega Institute for Medical Research, Laboratory of Virology and Chemotherapy, KU Leuven, Leuven, Belgium; 2 Faculty of Biological and Environmental Sciences, Molecular and Integrative Bioscience Research Programme, University of Helsinki, Helsinki, Finland; 3 Helsinki Institute of Life Sciences, Institute of Biotechnology, University of Helsinki, Helsinki, Finland; 4 Department of Pharmaceutical Sciences & Technology, Birla Institute of Technology, Mesra, Ranchi, Jharkhand, India; Stanford University, UNITED STATES

## Abstract

Rhino- and enteroviruses are important human pathogens, against which no antivirals are available. The best-studied inhibitors are “capsid binders” that fit in a hydrophobic pocket of the viral capsid. Employing a new class of entero-/rhinovirus inhibitors and by means of cryo–electron microscopy (EM), followed by resistance selection and reverse genetics, we discovered a hitherto unknown druggable pocket that is formed by viral proteins VP1 and VP3 and that is conserved across entero-/rhinovirus species. We propose that these inhibitors stabilize a key region of the virion, thereby preventing the conformational expansion needed for viral RNA release. A medicinal chemistry effort resulted in the identification of analogues targeting this pocket with broad-spectrum activity against Coxsackieviruses B (CVBs) and compounds with activity against enteroviruses (EV) of groups C and D, and even rhinoviruses (RV). Our findings provide novel insights in the biology of the entry of entero-/rhinoviruses and open new avenues for the design of broad-spectrum antivirals against these pathogens.

## Abstract 2

This study identifies a novel druggable pocket present in most enterovirus and rhinovirus particles, using this to develop broader-spectrum antivirals that inhibit enteroviruses from different groups, probably by interfering with viral RNA release.

## Introduction

Rhino- and enteroviruses (family Picornaviridae) are important human pathogens. Rhinoviruses are responsible for the common cold and are the most important trigger of exacerbations of asthma and chronic obstructive pulmonary disease (COPD) [1]. Each year, millions of people are affected by enteroviruses; there are 29 Coxsackieviruses, 28 echoviruses, and 5 other enteroviruses that cause disease (such as hand, foot and mouth disease, myocarditis, pancreatitis, aseptic meningitis, and encephalitis) in man [2]. In recent years, massive outbreaks of the enterovirus 71 (EVA71) occurred in Asia that left a large number of children with life-threatening encephalitis [3]. In 2014 and 2016, widespread outbreaks in the United States of the enterovirus 68 (EVD68) resulted in severe respiratory illness in children that was in many cases associated with acute flaccid myelitis, a condition now documented across the world in 14 countries [4]. Poliovirus (PV) is yet another important enterovirus. An enormous effort by the Global Polio Eradication Initiative (whereby 2.5 billion children have been vaccinated, requiring a $14 billion investment) has been very successful in massively reducing the number of cases [5]. However, the polio endgame appears to be much more challenging than anticipated; it is now well accepted that antivirals will be needed for a successful eradication, in particular to control outbreaks of circulating vaccine-derived polioviruses (cVDPV) and to stop excretion in chronic shedders (www.polioeradication.org). There are, however, no antivirals available for the treatment and/or prophylaxis of infections with entero- (including polio) and/or rhinoviruses. The best studied inhibitors are the so-called “capsid binders” (such as pleconaril, pirodavir, vapendavir, and pocapavir). These compounds fit in a hydrophobic pocket in the viral particle, thereby preventing binding of the virus to the receptor and/or uncoating [6]. Capsid binders have the disadvantage that they select rapidly for drug-resistant variants; this was also recently documented when assessing the effect of pocapavir in healthy volunteers challenged with the oral live attenuated polio vaccine virus [7]. Moreover, some capsid binders, such as pleconaril, lack activity against certain enteroviruses such as EVA71 [8]. Also, rhinoviruses of group C (infections with these viruses are associated with severe respiratory infections and childhood asthma exacerbations) are completely resistant to the capsid binders because the hydrophobic pocket is collapsed in the particle [9]. Today, the hydrophobic pocket is the only druggable surface pocket of the entero-/rhinoviruses particle. Now 32 years after its discovery, we report on a novel druggable pocket within a conserved VP1-VP3 interprotomer interface of the viral capsid. We propose that this pocket is crucial for conformational changes required for viral RNA release, and we describe a class of compounds targeting this pocket with broad-spectrum activity against entero-/rhinoviruses.

## Results

### An early-stage inhibitor of viral replication

**Fig 1:**
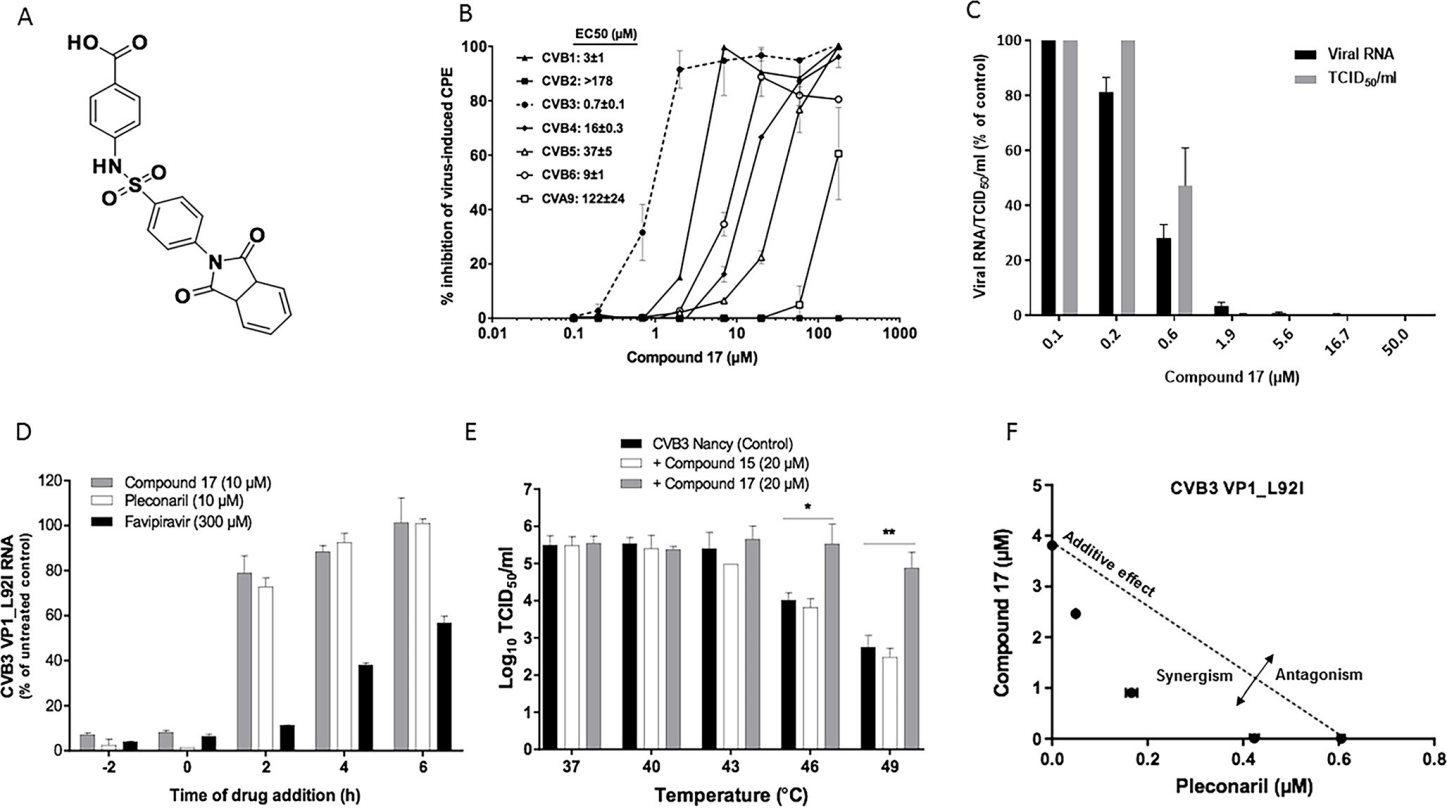
Compound 17 is an early-stage inhibitor of CVB3. (A) Compound 17’s formula. (B) Dose-response antiviral activity of compound 17 on the replication of group B enteroviruses in a CPE reduction assay. Data are mean values ± SD of three independent experiments. (C) Validation of the dose-response effect of compound 17 on CVB3 virus yield (viral RNA and infectious viral particles). Data are mean values ± SD of three independent experiments. (D) Time-of-drug-addition assay. Data are mean values ± SD of at least two independent experiments. (E) Thermostability assay in the presence or absence of compound 17 or the inactive analogue, compound 15. Values are the mean ± SD of three independent experiments. *p < 0.05, **p < 0.01 by unpaired *t* test. (F) Combination study of compound 17 with pleconaril. The graph is a plot of combination indices (CIs) versus the EC_50_ values of compounds at different combination ratios. Data are mean values ± SD of two independent experiments. The raw data of figures are presented in **S1 raw data**. CI, combination index; CPE, cytopathic effect; CVB, Coxsackievirus B; EC_50_, 50% effective concentration; TCID_50_, 50% tissue culture infective dose.

A benzene sulfonamide derivative, compound 17 (**Fig 1A**) was identified in a cell-based antiviral screening as a potent in vitro inhibitor of the Coxsackievirus B3 (CVB3) Nancy strain (50% effective concentration [EC_50_] 0.7 ± 0.1 μM; **Fig 1B**). The molecule has no adverse effect on the cells up to a concentration of 296 μM. Compound 17 also inhibits the replication of CVB1 and CVB6, and exerts moderate activity against CVB4, CVB5, and Coxsackievirus A9 (CVA9), but is devoid of activity against CVB2 at the highest concentration tested **(Fig 1B)**. CVA16 and EVA71 (EV-A group), CVA21 and PV1 (EV-C group), EVD68 (EV-D group), and Rhinovirus B14 (RVB14, RV-B) are not inhibited by the compound (**S1 Table**). The antiviral activity of compound 17 was further confirmed in a virus yield assay in which it reduced, in a dose-dependent manner, the production of CVB3 RNA and CVB3 infectious virus particles with EC_50_ values of 0.4 ± 0.01 μM and 1.1 ± 0.3 μM, respectively **(Fig 1C)**. Because the CVB3 Nancy strain has a substitution of leucine for isoleucine at position 92 of VP1 that confers resistance to pleconaril-like compounds [10], a pleconaril-sensitive CVB3 Nancy variant (VP1_L92I) was engineered to compare the time-of-addition profile of compound 17 with that of pleconaril and favipiravir (a viral polymerase inhibitor) [11]. Similar to pleconaril, compound 17 was found to target CVB3 replication at an early stage of the viral cycle because most of the antiviral activity was lost when the compound was added 2 h postinfection **(Fig 1D)**. Early-stage enterovirus inhibitors, such as pleconaril, are known to interact with the viral capsids and to increase their stability to heat inactivation [12]. In contrast to untreated virus or virus treated with 20 μM inactive analogue (compound 15), incubation with compound 17 increased the thermostability of CVB3 by 1.5 and 2.1 log10 50% tissue culture infective dose per mL (TCID_50_/mL) at 46°C and 49°C, respectively **(Fig 1E).** This result is comparable with what has been previously reported for other capsid binders [12,13], indicating a direct binding/interaction between compound 17 and the viral capsid. When pleconaril and compound 17 were combined, a synergistic antiviral activity was observed, suggesting that they have different mechanisms of antiviral action **(Fig 1F)**.

### A VP1-VP3 interprotomer binding pocket

**Fig 2:**
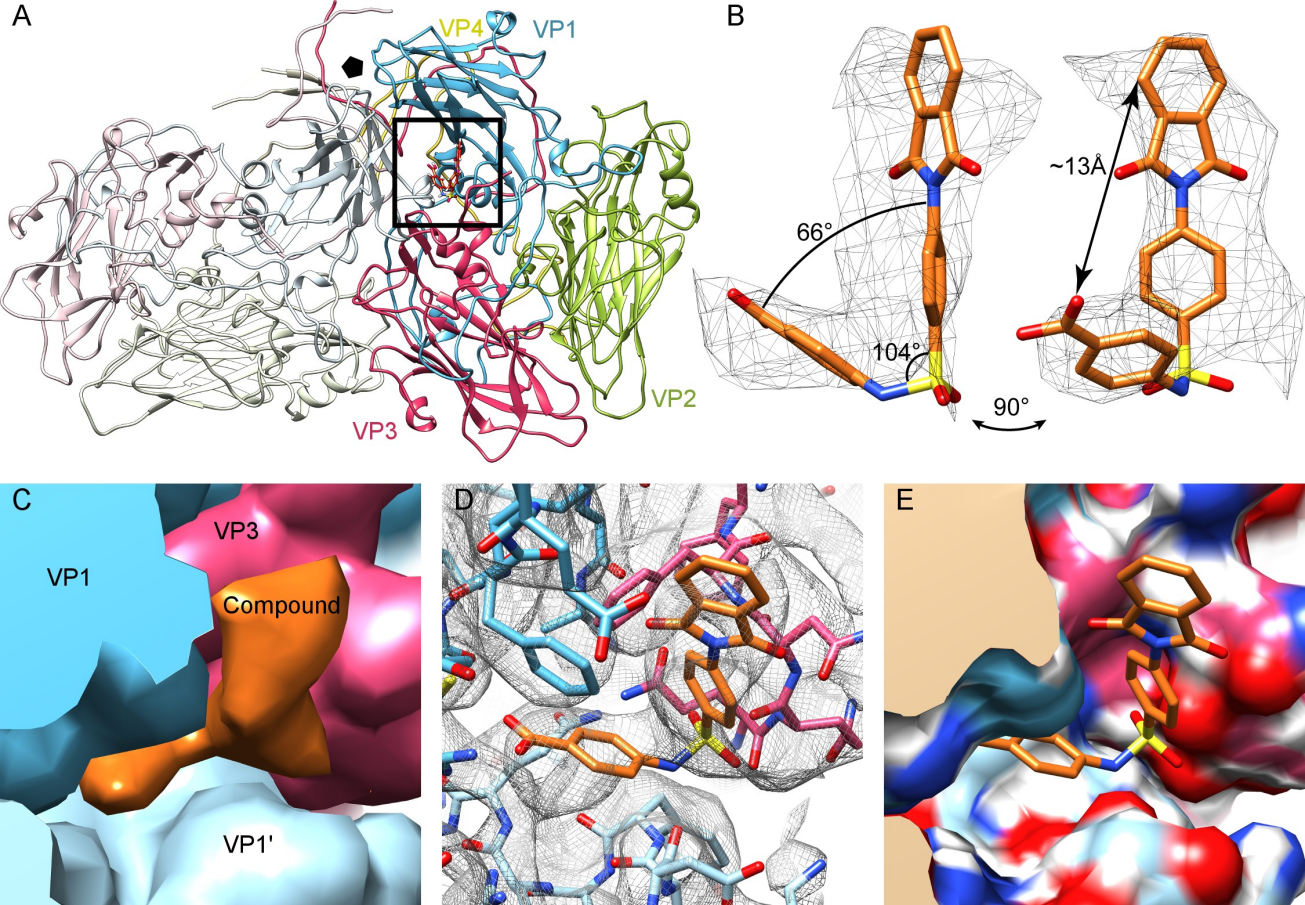
A novel druggable pocket of CVB3. (A) The atomic model of CVB3 Nancy in complex with compound 17 based on the cryo-EM density shows the position of the drug at an interprotomer site, located between adjacent VP1 chains (gray and blue) and VP3 (red). (B) Difference density for compound 17 (mesh), with an atomic model of compound 17 fitted in, shown at 1.5 standard deviations above the mean. (C) The process of difference mapping: simulated density map was generated from 1COV for the capsid proteins (here colored blue, light blue, red by protein) and normalized to the cryo-EM density map in UCSF Chimera. When the simulated density map of 1COV was then subtracted from the cryo-EM density, the difference density remained (orange). (D) Model docked into map. (E) Electrostatic analysis of the surface pocket. The raw cryoelectron microscopy data are deposited in the EMPIAR (Electron Microscopy Public Image Archive) database with the ID: EMPIAR-10199. CVB, Coxsackievirus B; EM, electron microscopy; UCSF, University California San Francisco.

To gain insights into the interaction of the drug with the viral capsid, a 4.0-Å structure of the CVB3–compound 17 complex was determined by cryo–electron microscopy (EM) and image reconstruction **(S1 and S2 Figs)**. Capsid protein side chains were readily identified and a CVB3 Nancy homology model could be flexibly fitted into the density map (**S2 Table)**. Unlike in the published CVB3 crystal structure (Protein Data Bank [PDB] ID: 1COV), the cryo-EM map of the Nancy strain lacks density for the lipid factor within the hydrophobic pocket of VP1 [14]. However, difference map analysis revealed additional density that could easily be fitted with an atomic model of compound 17 **(Fig 2A and 2C)**. The compound has an L-shaped structure with a longer and a shorter arm, allowing unambiguous docking **(Fig 2B)**. Modelling revealed that compound 17 binds into a pocket formed by two VP1 units and one VP3 unit at an interprotomer interface. Because of the icosahedrally symmetric nature of the virus, there are 60 such sites in one capsid, with 5 around each 5-fold vertex **(Fig 2C and S3 Fig)**. This interprotomer site is 16 Å away from the hydrophobic pocket targeted by pleconaril and other capsid inhibitors, raising the possibility of complementary antiviral effects (for those strains susceptible to both classes of compounds) **(S3 Fig)**. Modelling of the interaction within Proteins, Interfaces, Structures, and Assemblies (PISA) **(S3 Table)** revealed residues of VP1 (73, 75–78, 155–157,159–160, 219, and 234) and VP3 (233–236) that line the pocket, as shown in **Fig 2D and 2E**. From analyzing the conservation of these pocket residues in 56 CVB3 amino acid sequences retrieved from GenBank, 7 out of 16 residues appeared to be completely conserved, and, on average, 14 of the residues were very similar (>97% when assessed by Grantham distance) **(S3 Table)**. The pocket also shows conservation across the enterovirus B group, with 7 of 16 residues identical across the strains tested **(S3 Table)**, and a good retention of pocket shape, as assessed by root mean square deviation (RMSD) **(S4 Table).**

### Critical residues of the pocket

A clonal in vitro resistance selection [13] was performed to select compound-resistant virus variants. Four resistance mutations were identified mapping to the VP1 coding region of the genome. Three of the four amino acid mutations were located in the pocket vicinity: F76C, E78G, and A98V, and one in the 5-fold vertex region (D133G) **(Fig 2D, S4A Fig)**. Twelve reverse-engineered CVB3 mutants were generated in a CVB3 infectious clone (Nancy strain). These were selected based on (i) residues that line the pocket in the atomic model and (ii) mutations identified in the resistance selection to compound 17. In addition, the glutathione-independent VP1_T77M CVB3 variant was engineered (that is resistant to the glutathione-depleting compound TP0219) [15]. Of these mutants, eight proved viable **(Table 1 and Fig 2D)** and were further investigated, whereas four were not viable (VP1_Q160G and VP1_R234G, VP3_F236G and VP3_Q233G). Seven out of the eight variants proved resistant to compound 17 and were equally sensitive to the control compound, i.e., the 3C protease inhibitor rupintrivir **(Table 1)**. These results validate the importance of the pocket residues for compound 17 activity.

**Table 1:** Effect of compound 17, rupintrivir (a 3C-protease inhibitor), and TP0219 (a glutathione-depleting compound) on the in vitro replication of CVB3 WT and reverse-engineered CVB3 variants in CPE reduction assays.

Variant	Compound 17		Rupintrivir		TP0219	
	EC_50_ ± SD (μM)	RR	EC_50_ ± SD (μM)	RR	EC_50_ ± SD (μM)	RR
WT	0.7 ± 0.1	-	2.8 ± 0.5	-	16.7 ± 4	-
VP1_Y75C	7.9 ± 3.5	11**	ND	ND	ND	ND
VP1_F76C	12 ± 1	18***	2 ± 1	0.7	2.3 ± 1	0.14
VP1_T77M	15 ± 4	21***	ND	ND	>80	>5
VP1_E78G	15 ± 2	21***	2 ± 0.03	0.7	19 ± 4	1.1
VP1_A98V	2.2 ± 0.3	3**	3 ± 0.6	1.1	27 ± 6	1.6
VP1_D133G	28 ± 3	38***	3.4 ± 1	1.2	11 ± 1	0.7
VP1_D155G	>40	>57	ND	ND	ND	ND
VP3_N235G	1.0 ± 0.2	1.2	ND	ND	ND	ND

Data were obtained from at least three independent experiments.

*p < 0.005.

**p < 0.001.

***p < 0.0001 (unpaired *t* test).

Abbreviations: CPE, cytopathic effect; CVB, Coxsackievirus B; EC_50_, 50% effective concentration; ND, not determined; RR, relative resistance (EC_50_ of the mutant/EC_50_ of WT); WT, wild-type.

As for the only resistance mutation located outside the interprotomer pocket, the thermostability profile of the VP1_D133G variant revealed that it is more heat sensitive than the CVB3 Nancy (the mutation destabilizes the viral capsid) and that compound 17 could still stabilize the variant **(S4B Fig)**. Hence, VP1_D133G is a compensatory mutation rather than a mutation that prevents compound 17 binding. The growth kinetics and plaque phenotyping of the variant revealed that the mutation slightly decreases virus fitness **(S4C Fig).**

VP1_T77 has been implicated in glutathione binding [15], and we show here that the glutathione-independent VP1_T77M CVB3 variant has a reduced sensitivity to compound 17. Therefore, we assessed the impact of increasing intracellular glutathione on the antiviral activity of compound 17. The antiviral activity of compound 17 was unaffected by the glutathione ethyl ester, even at the highest concentration tested **(S5 Fig).**

We next assessed the potential effect of compound 17 on viral receptor binding. We used a flag-tagged human Coxsackievirus and adenovirus receptor (hCAR) protein **(S6A Fig)** bound to beads to pull down the virus in the presence or absence of compound 17. The binding affinity of CVB3 was not reduced in the presence of compound 17, even at the highest concentration tested **(S6B Fig)**. Moreover, compound 17 was active against a decay-accelerating factor (DAF)–dependent enterovirus B, E-11 virus **(S6C Fig)**. Neither of the two reported binding sites of Coxsackievirus and adenovirus receptor (CAR) and DAF on CVB3 (PDB ID:1JEW [16]; PDB ID:3J24 [17]) overlap with the interprotomer pocket, which further corroborates the experimental findings.

### Developing a broad-spectrum inhibitor

**Fig 3:**
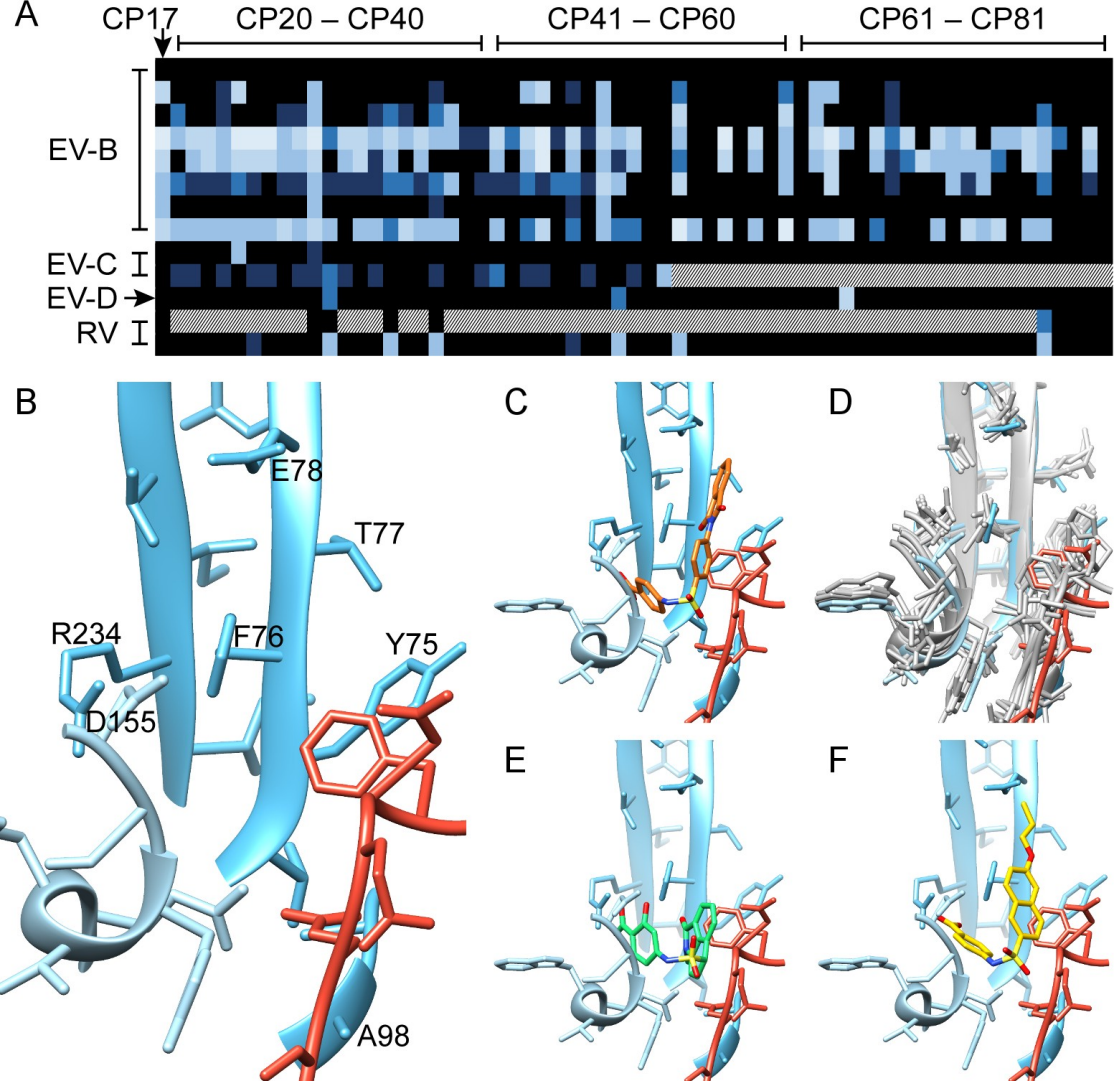
Discovery of new CP17 analogues with improved potency and spectrum. (A) Heat map of the activity (EC_50_) of CP17 and the 62 analogues (CP20–CP81) in the context of EV-B (CVB1, CVB2, CVB3, CVB3_L92I, CVB4, CVB5, and CVB6), EV-C (CVA21 and PV1), EV-D (EVD68), and RV (RVA63 and RVB14) infection. Color range: black (inactive compounds)–light blue (compounds with EC_50_ < 1 μM). For white-dotted pixels, EC_50_ was not determined. EC_50_ values and compounds structures are available in S1 Table. (B) Pocket residues of VP1 proteins (blue, light blue) and VP3 (red), with those labelled that, when mutated, induced resistance to compound 17. (C) Position of compound 17 in the pocket (orange). (D) Pocket structural conservation for related enteroviruses exhibiting sensitivity to compound 17: an overlay of the solved structure (blue/light blue/red) with atomic models (gray) of CVB3 (wwPDB ID:1COV), CVA9 (wwPDB ID:1D4M), EVD68 (wwPDB ID:4WM8), E1 (wwPDB ID:1EV1), E7 (wwPDB ID:2X5I), and E11 (wwPDB ID:1H8T). RMSD statistics found in S4 Table. (E) The predicted docking of compound 29 (green) into the solved structure. (F) The predicted docking of compound 48 (yellow). CP17, compound 17; CVA, Coxsackievirus A; CVB, Coxsackievirus B; E, echovirus; EC_50_, 50% effective concentration; EV-B, enterovirus-B; EV-C, enterovirus-C; EV-D, enterovirus-D; EVD68, enterovirus D68; PV, poliovirus; RMSD, root mean square deviation; RV, rhinovirus; RVA63, rhinovirus A63; RVB14, rhinovirus B14; ww, worldwide.

The structural and interaction information of compound 17 with the discovered pocket led to the identification of the core structure of this compound **(S7 Fig)**. In a first step for hit optimization, commercially available analogs (compounds 20–50) with different R1 groups were acquired. Next, derivatives with different R2 and R3 modifications were obtained through medicinal chemistry efforts (compounds 51–81). Specifically, this was an iterative process whereby information from the antiviral assays was used to guide the synthesis of more active/selective analogues. This resulted in analogues with activity against enteroviruses belonging to different groups, including EV-B (CVBs), EV-C (PV1 and CVA21), EV-D (EVD68), RV-A (RVA09, RVA59, and RVA63), and RV-B (RVB14) species **(Fig 3A and S1 Table)**. None of the tested compounds was active against EVA viruses (CVA16 and EVA71). Selected compounds, which exerted the most potent activity for each virus, are presented in **Table 2** (the structural formulae are presented in **S1 Table**). Among the analogues, compounds 29 and 48 resulted in activity against all the six CVBs tested **(Table 2, S8 Fig)**. Compound 48 also completely inhibited PV1 replication at concentration of 144 μM **(Table 2, S8 Fig)**. Another analogue, i.e., compound 77, elicited antiviral activity against RV-A and RV-B species **(Table 2)**. The shape of the new molecules in the series is complementary to that of the pocket **(Fig 3)**. A clear structure-activity relationship (SAR) was deduced from the carboxylic group at the R3 position, where a hydrogen atom was essential for antiviral activity, and at position R2, where a hydroxyl group proves overall beneficial for broad antiviral action. The cryo-EM structure indicates that there is charge complementarity between R3 and the conserved residue VP1_R234. Sterically, this is also a restricted region of the pocket, preventing bulky R3 groups (Fig 2C–2E). A superposition of the viral structures available for susceptible strains revealed a significant conservation of the interprotomer pocket, which is in line with the number of pan-enterovirus active analogues identified in this first screen **(Fig 3, Table 2, S1 Table)**. Based on the structural alignment of the pocket in different enteroviruses, we predicted that some of the compounds in this class would be also active against echoviruses 1 and 7 (E-1 and E-7). Subsequent antiviral assays confirmed the prediction and suggest that computational analysis will be instrumental for the development of more potent and broad-spectrum analogues **(Fig 3, Table 2,** and **S4 Table)**.

**Table 2:** Hit expansion of compound 17.

Compound	EC_50_ ± SD (μM)														
ID	CVB1	CVB2	CVB3	CVB4	CVB5	CVB6	E-1	E-7	PV1	CVA21	EVD68	RVA09	RVA59	RVA63	RVB14
**17**	**3.1 ± 0.8**	**>296**	**0.7 ± 0.1**	**16 ± 0.3**	**37 ± 5**	**9 ± 0.5**	**10 ± 3**	**5.2 ± 0.7**	**>400**	**>296**	**>296**	**>296**	**>296**	**>296**	**>296**
24	7.4 ± 4.6	>480	0.6 ± 0.1	61 ± 15	>480	12 ± 0.3	ND	ND	277 ± 72	38 ± 3	>480	ND	ND	ND	>480
25	461 ± 51	>480	0.3 ± 0.1	155 ± 62	>480	20 ± 4	ND	ND	316 ± 2	>480	>316	ND	ND	ND	119 ± 13
29	16 ± 10	14 ± 0	0.4 ± 0.1	21 ± 5	12 ± 2	2.0 ± 0.02	4.3 ± 0.1	39.8 ± 0.3	307 ± 30	203 ± 17	>400	ND	ND	ND	>400
30	>450	>450	71 ± 31	281 ± 9	>450	15 ± 2	ND	ND	>450	>450	79 ± 4	ND	ND	ND	25 ± 2
48	29 ± 0.4	15 ± 2	8.2 ± 0.2	8.6 ± 0.8	29 ± 5	15 ± 6	24 ± 0.9	21 ± 2	27 ± 1	>197	>64	ND	ND	ND	>64
53	85 ± 6	4 ± 0.3	11 ± 3	9.4 ± 1.3	>300	1 ± 0.1	ND	ND	ND	>300	>107	ND	ND	ND	13 ± 1
60	67 ± 9	8.4 ± 1.1	12 ± 3	12 ± 2	>250	0.2 ± 0.1	ND	ND	ND	>250	>75	ND	ND	ND	>75
63	35 ± 5	>400	0.9 ± 0.2	6.1 ± 1	>400	22 ± 3	ND	ND	ND	>400	>400	ND	ND	ND	>400
64	>250	>250	36 ± 7	>250	>250	3.8 ± 0.5	ND	ND	ND	>250	8.7 ± 0.6	ND	ND	ND	>63
77	>250	98 ± 3	11 ± 4	59 ± 5	>250	18 ± 1	ND	ND	ND	>250	>174	118 ± 4	100 ± 2	99.0 ± 0.2	45 ± 8

Data ± SD are mean values for at least two independent experiments.

Abbreviations: CVA, Coxsackievirus A; CVB, Coxsackievirus B; E, echovirus; EC_50_, 50% effective concentration; EVD68, enterovirus D68; ND, not determined; PV1, poliovirus type 1; RVA, rhinovirus A; RVB, rhinovirus B.

## Discussion

Pockets traditionally make more attractive targets for drug design and optimization than flat surfaces, as they can accommodate larger surface areas and hence contribute more residues for interaction. For the same reason, pockets such as the hydrophobic site found within VP1 of most enteroviruses play important functional roles during virus entry and replication. Until now, the hydrophobic canyon site has been the only explored surface pocket for enteroviruses. The cryo-EM structure of CVB3 in complex with compound 17 revealed that 16 residues line the interprotomer pocket and provided a plausible explanation for most of the observed resistance mutations identified by selection. To confirm the druggable site, several of the residues were mutated, and the resulting viruses had indeed reduced sensitivity to compound 17. There were several amino acid residues in the pocket that could not be mutated, highlighting the importance of this pocket in the biology of enterovirus replication. Residues in the pocket allow quaternary structural changes in the capsid as it progresses from the virion to an expanded altered particle (A-particle) during entry, primed for RNA release [2,18], a process common to many picornaviruses [2]. This expansion requires rotation and translation of VP1, relative to itself, and to VP3 and VP2, a process dependent on movements along the interprotomer interfaces, including the region containing the pocket described here. Comparison of several structures of expanded picornavirus particles revealed that in the expanded particle, the pocket has an altered shape caused by the movement of the VP3 C terminus; this includes the residues that line the pocket (Q233, Q234, N235, F236). Hence, we propose that binding of compounds in this pocket stabilizes the conformation of a key region of the virion, preventing rearrangements that allow transition to the A-particle. The resistant mutant distant to the pocket (VP1-D133G) destabilizes the particle sufficiently so that the energy barrier to the transition is lowered, compensating for the increased stability induced by the drug, and thus still allowing release of the RNA.

To conclude, compound 17 and its analogues are selective inhibitors of CVB3 replication that target a novel pocket on the surface of the capsid that is important in conformational changes required for RNA release from the viral particle. Cryo-EM structural data and in vitro resistance selection together with reverse engineering revealed a set of amino acid residues in the virion that are crucial for the compound activity and allowed us to propose a unique mechanism of antiviral action. Antiviral evaluation of a set of compound 17 analogues indicates that it is possible to target EV-B, EV-C, EV-D, and even rhinovirus (A and B) species. In addition, computational analysis suggests that the pocket is partially conserved across species, including the RV-C species that are naturally resistant to pleconaril (and molecules with a similar mechanism of action) and EV-A. However, the shape and electrostatic properties of the pocket are sufficiently different in those latter species so that further study is required to design molecules that also block viruses belonging to these species **(S3 and S4 Tables)**. Medicinal chemistry efforts are ongoing to replace the carboxylic acid moiety in the core structure of this class of compounds with more stable moieties, such as (bio)isosteres. Hence, we foresee the development of a future series of analogues with improved activity and spectrum against an important group of viruses that cause significant infectious illnesses worldwide.

## Material and methods

### Cells and viruses

African green monkey kidney (Vero A, ATCC CCL-81) cells, Buffalo green monkey (BGM, ECACC 90092601) cells, HeLa Rh cells (kindly provided by Dr. K. Andries [Janssen Infectious Diseases, Beerse, Belgium]), and human rhabdosarcoma (RD, ECACC 85111502) cells were maintained in minimal essential medium (MEM Rega-3, Gibco, Belgium) supplemented with 10% fetal bovine serum (FBS, Gibco, Belgium), 2 mM l-glutamine (Gibco, Belgium), and 1% sodium bicarbonate (Gibco, Belgium). Antiviral assays medium was supplemented with 2% FBS instead of 10%. All cell cultures were maintained at 37°C in an atmosphere of 5% CO_2_ except for rhinovirus 14, which was incubated at 35°C. Coxsackievirus B3 strain Nancy (CVB3, derived from plasmid P53CB3/T7), Coxsackievirus B1 (Conn-5), Coxsackievirus B2 (CVB2, OH), Coxsackievirus B5 (CVB5, Dekking), Coxsackievirus B6 (CVB6, stam P2183), enterovirus 71 (EVA71, BrCr), and enterovirus 68 (EVD68, CU70) were kind gifts from Prof. F. van Kuppeveld (Universiteit Utrecht, Utrecht, the Netherlands). Coxsackievirus B4 (CVB4, Edwards) was kindly provided by Dr. J.W. Yoon (Julia McFarlane Diabetes Research Centre, Canada). The Sabin vaccine strain of PV1 was kindly provided by Dr. A.J. Macadam (Centre for Infectious Diseases, University of Edinburgh, Edinburgh, United Kingdom). Human rhinovirus type 14 (RVB14) and echovirus 11 (ECHO11) was kindly provided by Dr. K. Andries (Janssen Infectious Diseases, Beerse, Belgium).

### Compounds

Compound 17 and its inactive analogue compound 15 (where the carboxyl group has been replaced with a methyl group) were synthesized as described previously [19]. Favipiravir (T-705) was purchased as a custom synthesis product from BOC Sciences (NY). Rupintrivir was purchased from Axon Medchem (the Netherlands). Pleconaril was kindly provided by V. Makarov (RAS Institute of Biochemistry, Russia). All compounds were dissolved in analytical grade DMSO (10 mg/mL).

### Cytopathic effect reduction assay

Cells were seeded (at a density of 2.5 × 10^4^ cells/well for Vero A, BGM cells, and RD cells and 1.8 × 10^4^ cells/well for HeLa Rh cells) in 96-well tissue culture plates (BD Falcon) and were allowed to adhere overnight. The next day, cells were treated with serial dilutions of the compound and infected with the selected virus strain at a multiplicity of infection (MOI) of 0.01. On days 2–3 postinfection, the antiviral effect of the compound was quantified using the 3-(4,5-dimethylthiazol-2-yl)-5-(3-carboxymethoxyphenyll-2-(4-sulfophenyl)-2-H-tetrazolium, inner salt)/phenazine methosulfate (MTS/PMS) assay, as described by the manufacturer Promega (the Netherlands). In addition, the cells were also checked microscopically for minor signs of virus-induced cytopathic effects (CPE) or compound-induced toxicity (changes in cell and/or monolayer morphology). The EC_50_, which is the concentration of compound that is required to inhibit virus-induced cell death by 50%, was determined using logarithmic interpolation.

### Virus yield assay

Vero A cells were seeded at a density of 5 × 10^4^ cells/well in 96-well tissue culture plates. The next day, cells were treated with serial dilutions of the compound and then infected with CVB3 (MOI, 0.01). After a 2-h incubation, the cells were washed three times with assay medium to remove non-adsorbed virus. The cells were treated with the same serial dilutions of the compound as for the CPE reduction assay and incubated for 2 days at 37°C. At the end of the incubation period, the viral RNA was isolated from the culture supernatants in different wells using NucleoSpin 96 virus kit (Macherey Nagel, Germany) and was quantified by real-time quantitative reverse transcription PCR (qRT-PCR) (see below). In addition, the number of infectious virions in the collected supernatants was determined by an end-point titration assay, as described by Reed and Muench (TCID_50_/mL) [20].

### qRT-PCR

For each RT-PCR reaction, 25 μL of a PCR reagent mixture was prepared to contain 12.5 μL One-Step qRT-PCR mix (Eurogentec, Seraing, Belgium), a 900 nM concentration of each primer, a 200 nM concentration of the specific TaqMan probe, RNase-free water, and 5 μL RNA. The nucleotide sequences of the used primers and probe were as follows: forward primer (5′-ACG AAT CCC AGT GTG TTT TGG-3′), reverse primer (5′-TGC TCA AAA ACG GTA TGG ACA T-3′), and CVB3 probe (5′-FAM-CGA GGG AAA CGC CCC GCC-TAMRA-3′). The RT-PCR reaction was done using the ABI 7500 Fast Real-Time PCR System (Applied Biosystems, Branchburg, NJ) according to the following protocol: 30 min at 48°C and 10 min at 95°C, followed by 40 cycles of 15 s at 95°C and 1 min at 60°C. The RNA copy number in each sample was determined by a standard curve generated using serial dilutions of CVB3 standard cDNA that were included in the run [21].

### Time-of-drug-addition assay

Vero-A cells were seeded in 96-well tissue culture plates at a density of 5 × 10^4^ cells/well in assay medium and incubated overnight. Cells were treated with the appropriate concentration of the compound before or after the infection with CVB3 VP1_L92I variant (pleconaril sensitive, MOI = 1) at the following time points (−2 h, zero time, 2 h, 4 h, and 6 h). Following 24 h of incubation, the intracellular viral RNA from treated and untreated cells was extracted using Cells-to-cDNA lysis buffer (Life Technologies, the Netherlands) and was quantified by quantitative RT-PCR, as described before.

### Thermostability assay

CVB3 WT (5 × 10^5^ TCID_50_/mL) was incubated with 20 μM compound 17, 20 μM compound 15 (an inactive analogue) [22], or an equal volume of assay medium) at six different temperatures ranging from 37–52°C for 2 min, followed by rapid cooling to 4°C. Subsequently, the infectious virus load of the different samples was quantified by end-point titration.

### Large-scale virus purification

For large-scale virus purification for structural study, CVB3 Nancy strain was grown on Vero A cells, maintained in MEM (Eagle, without L-glutamine, Sigma M2279) supplemented with sodium bicarbonate (1%), Glutamax (2 mM, Gibco, 35050–038), antibiotic/antimycotic solution (1%, Sigma, A-5955), and 10% FBS. For proliferation, 30× T175 flasks with confluent cell layers (approximately 90%) were inoculated with CVB3 Nancy strain at an MOI of 0.02 in serum-free medium. After CPE was observed over the entire cell monolayer, the contents of the flask were collected, freeze-thawed three times, and the lysate cleared by centrifugation (3,000*g*, 277 K, 15 min). The supernatant was filtered and concentrated by ultrafiltration in Centricon 100-kDa filter units (Millipore). Ultracentrifugation was then performed on a CsCl step gradient, for which the top density was 1.25 g/cm^3^ and the bottom 1.48 g/cm^3^ CsCl (32,000*g*, Beckmann SW41Ti, 277 K, 18 h), in buffer (0.01 M HEPES, pH 7.5, 0.1 M MgCl_2,_) with additional 0.5% CHAPS detergent [23]. The virus band was collected, and the CsCl and CHAPS removed in a buffer exchange by ultrafiltration in Centricon 100-kDa filters (Millipore). Ultracentrifugation, band collection, and buffer exchange steps were performed twice.

### Cryo-EM

Purified CVB3 was mixed with compound 17 at a 1:2,500 molar ratio of capsid to inhibitor and incubated for 1 h at 37°C prior to cryo sample preparation on glow-discharged ultrathin carbon on lacey carbon grids (#01824, Ted Pella, US). Micrographs were collected at the Diamond Light Source, UK, via iNEXT access using a FEI Titan Krios electron microscope equipped with a Gatan energy filter (post-GIF) and a Gatan K2 Summit direct electron detector. The K2 detector was operated in counting mode, recording movies at a nominal magnification of 130,000× (sampling 1.06 Å/pixel), using a total electron dose of approximately 47 e/Å^2^ separated into 20-dose fractions. Example micrographs are shown in **S1A Fig.**

### Image processing

For each movie collected, all 20-dose fractions were aligned and compensated for drift using MotionCor2 [24]. The defocus level and other CTF parameters of each micrograph were determined using GCTF [25]. Micrographs were discarded if the power spectrum indicated noticeable astigmatism, crystalline ice, or uncorrected drift. For each of the remaining 2,121 movies, 71,034 particles were automatically picked on aligned averaged images using Relion 2.0.4 [26]. Two-dimensional classification was used to select 10,182 particles for further processing. The starting model was generated by Fourier filtering a CVB3 capsid atomic model (PDB ID: 1COV) [14] to a resolution of 40 Å in UCSF Chimera [27]. Three-dimensional classification in Relion [28] was used to remove empty, broken, and overlapping particles. All 20 frames were used for classification, refinement, and calculation of the final density map in Relion 2.1 beta from 4,891 particles. The final refinement step combining two independent data sets gave a resolution of 4.0 Å, as assessed by 0.143 criterion Fourier shell correlation [29]. A B-factor of −170 Å^2^ was automatically calculated in Relion and applied. The reconstruction central section is shown in **S1B Fig**, and resolution estimates are shown in **S2 Fig.**

### Atomic model building and refinement

An initial atomic model for the Nancy strain CVB3-inhibitor complex was generated using I-TASSER and UCSF Chimera [27,30]. I-TASSER threaded the Nancy CVB3 sequence onto the crystal structure of the CVB3 coat protein (PDB ID: 1COV [14]). UCSF Chimera built compound 17 atom by atom from the simplified molecular-input line-entry system (SMILES) line notation that described the structure of the chemical species. Docking of atomic coordinates was done manually using UCSF Chimera and the fit was further optimized using the “Fit in Map” command. Inspection and further refinement of the viral atomic coordinates was done using Coot, and this served as input for molecular dynamics flexible fitting (MDFF) [31]. The MDFF program was used together with NAMD and VMD to further enhance the fit of the model into the cryo-EM density [32–34]. A scale factor of 1 was employed to weight the contribution of the cryo-EM map to the overall potential function used in MDFF. Simulations included 20,000 steps of minimization and 100,000 steps of molecular dynamics under implicit solvent conditions with secondary structure restraints in place. Model statistics are shown in **S2 Table.** Interface analysis was done with the PDBePISA www server [35] and with Coot [31] **(S3 Table)**. RMSD analysis of the pocket conservation was calculated in UCSF Chimera **(S4 Table)**.

### Isolation of compound 17–resistant CVB3 variants and reverse genetics

A multistep clonal selection protocol was performed to obtain compound 17–resistant CVB3 variants [13]. Whole-genome sequencing was performed to detect any mutations in the genome of the putative compound-resistant variants (3130 Genetic Analyzer automatic sequencer, Applied Biosystems). Desired mutations were introduced into the CVB3 strain Nancy infectious cDNA clone kindly provided by Prof. F. van Kuppeveld (University of Utrecht, Utrecht, the Netherlands) using the QuikChange mutagenesis kit (Agilent, CA) according to the manufacturer’s instructions. The presence of the desired mutation in the produced construct was confirmed by sequencing. Full-length infectious viral RNA was then transcribed from each plasmid using the RiboMAX Large Scale RNA Production System-T7 (Promega, the Netherlands) and transfected into Vero A cells with a TransIT-mRNA Transfection Kit (Mirus) to produce virus stocks of the reverse-engineered mutants.

### Growth kinetics

The in vitro growth kinetics of CVB3 WT and VP1 variants were determined in Vero A cells (96-well tissue culture plates, 5 × 10^4^ cells/well). At time zero, cells were infected with the respective virus variants (MOI = 1). After 2 h of infection, the cells were washed three times with 2% medium to remove non-adsorbed virus, and 200 μL medium were added to each well. At 8 and 24 h postinfection, the culture supernatants were collected to quantify the number of infectious virus particles at each time point by end-point titration.

### Plaque phenotyping

Vero A cells were seeded in 6-well plates (BD Falcon 6-Well Cell Culture Plate) at a density of 1 × 10^6^ cells/well. The next day, cells were infected with 1 mL of 10-fold serial dilutions of the respective virus variants in assay medium. At 2 h postinfection, the cell monolayers were washed three times with PBS, after which 3 mL of a freshly prepared 1:1 mixture of 1% low-melting-point agarose (Invitrogen, Belgium) and 2× MEM medium (Gibco, Belgium) was added. After 3 d of incubation at 37°C, cells were fixed with 3.7% formaldehyde followed by removal of the agarose overlay and staining with 2% Giemsa staining solution to visualize the virus-induced plaques.

### Expression of a flag-tagged CAR

The pcDNA3.1-hCAR plasmid was kindly provided by Prof. F. van Kuppeveld (Universiteit Utrecht, Utrecht, the Netherlands). A flag tag was added to the C terminus of an hCAR sequence by fusion PCR using the following set of primers: forward, GAAAAGCTTCCACCATGGCGCTCCTGCTGTGC; reverse, GGA TCC CTA CTT GTC GTC ATC GTC TTT GTA GTC TAC TAT AGA CCC ATC CTT GCT. The primers were designed to add HindIII and BamHI restriction sites at the N and C termini of the hCAR sequence, respectively. Following PCR amplification, the hCAR-flag fragment was digested with HindIII and BamHI restriction enzymes and cloned back into pcDNA3.1 vector (that was digested with the same enzymes). The pcDNA3.1-hCAR-flag plasmid was then transfected into HEK293T cells in 6-well plates (7 × 10^5^ cells/well) using Mirus TransIT-LT1 Transfection Reagent according to the manufacturer’s protocol. The expression of flag-tagged hCAR protein on day 2 post-transfection was checked by immunostaining using ANTI-FLAG antibody (Sigma-Aldrich) rabbit IgG as a 1ry antibody and Alexa Fluor 488 goat anti-rabbit (Thermo Fisher Scientific, Belgium) as a secondary antibody. Fluorescence was detected using the FLoid Cell Imaging Station (Thermo Fisher Scientific, Belgium).

### Testing the effect of compound 17 on the binding of CVB3 to the CAR

The lysate from HEK293T cells transfected with a flag-tagged pcDNA3.1-hCAR plasmid was incubated with ANTI-FLAG M2 Magnetic Beads (Sigma-Aldrich) at 4°C overnight. After washing with PBS, the CAR-coated beads were incubated with CVB3 or mixtures of the virus with different concentrations of compound 17 for 2 h at 37°C. At the end of incubation, the beads were washed three times with PBS followed by elution of CAR-bound virus using RLT lysis buffer (Qiagen, Germany). The viral RNA in each sample was then extracted using an RNeasy kit (Qiagen, Germany) and quantified by real-time quantitative RT-PCR as described before [21].

## Supporting information

S1 Fig Microscopy and reconstruction. (A) An example micrograph from data collection processed for this paper. (B) Grayscale central section of the reconstruction. (C) Representative electron density and model fit for selected residues in VP2.
(TIF)

S2 Fig Reconstruction resolution estimates. (A) Estimation of local resolution of reconstruction using ResMap (Kucukelbir and colleagues, 2014, PMID: 24213166), showing the capsid protein at approximately 4 Å on unsharpened full map and central section. (B) Fourier shell correlation calculated in Relion, with a resolution estimate of 4.0 Å as assessed at the 0.143 criterion.
(TIF)

S3 Fig CP17 nestles into a pocket that differs from the one described for pleconaril. The hydrophobic pleconaril drug-binding pocket of the VP1 β-sandwich is shown in the Gauntt strain (left) with the pocket factor present (PDB ID: 3jd7), which is missing from the solved Nancy strain (right). Pleconaril enters this hydrophobic pocket and displaces the pocket factor. CP17, in contrast, targets a region on the outside of the VP1 β-sandwich (modeled into both strains). CP17, compound 17.
(TIF)

S4 Fig The drug-resistant variant, VP1_D133G, maps to the 5-fold vertex region. (A) Map of the VP1 residues involved in compound 17 resistance: residues F76, E78, and A98 map to the binding pocket identified using cryo-EM, and D133 is located in the central ion channel at the 5-fold vertex regions. (B) Thermostability assay: a high-titered stock of CVB3 VP1_D133G variant was incubated at different temperatures in the presence or absence of 20 μM compound 17. The residual infectivity of the virus was determined by end-point titration. Values are the mean ± SD of three independent experiments. Statistical differences (*p < 0.05, **p < 0.01) were analyzed by the unpaired *t* test. (C) Growth kinetics and plaque phenotyping of VP1_D133G variant: the infectious virus titer of CVB3 WT and VP1_D133G variant at different time points was determined by end-point titration. The plaque phenotype was determined by infecting Vero A cells with 10-fold serial dilution of each virus stock followed by addition of an agarose overlay. On day 3 postinfection, the viral plaques were visualized by Giemsa staining. The raw data of figures are presented in **S1 raw data**. CVB, Coxsackievirus B; WT, wild-type.
(TIF)

S5 Fig Effect of glutathione on the antiviral activity of compound 17. Effect of GEE on the antiviral activity of compound 17. Vero A cells were treated with 2-fold serial dilutions of the GEE (highest concentration 10 mM). Following 1 h of incubation, a fixed concentration of compound 17 (5 μM) or TP0219 (50 μM) was added to the GEE-treated and non-treated cells, followed by infection with CVB3 WT at an MOI of 0.01. On day 3 postinfection, the effect of GEE treatment on the antiviral activity of the tested compounds was quantified using the MTS/PMS method. Data represented are percentages of untreated controls and are mean values ± SD of three independent experiments. The raw data of figures are presented in **S1 raw data**. CVB, Coxsackievirus B; GEE, glutathione ethyl ester; MOI, multiplicity of infection; MTS/PMS, 3-(4,5-dimethylthiazol-2-yl)-5-(3-carboxymethoxyphenyll-2-(4-sulfophenyl)-2-H-tetrazolium; WT, wild-type.
(TIF)

S6 Fig Compound 17 does not interfere with the binding of CVB3 to the CAR. (A) Immunofluorescence image for expression of C-terminal flag-tagged CAR in HEK239T cells. (B) Immunoprecipitation of CVB3 with flag-tagged CAR in presence or absence of compound 17 as quantified by qRT-PCR. (C) In vitro antiviral activity of compound 17 against E-11 (a DAF-dependent enterovirus B) in a CPE reduction assay. The raw data of figures are presented in **S1 raw data**. CAR, Coxsackievirus and adenovirus receptor; CPE, cytopathic effect; CVB, Coxsackievirus B; DAF, decay-accelerating factor; E-11, echovirus 11; qRT-PCR, quantitative reverse transcription PCR.
(TIF)

S7 Fig Core structure derived from compound 17. (TIF)

S8 Fig **Dose-response antiviral activity of (A) compound 29 and (B) compound 48 on the replication of selected enteroviruses in a CPE reduction assay.** Data are mean values ± SD of at least two independent experiments. The raw data of figures are presented in **S1 raw data**. CPE, cytopathic effect.
(TIF)

S1 Table Effect of compound 17 and selected analogues on the replication of various enteroviruses. (DOCX)

S2 Table Details of atomic model and model refinement statistics for the CVB3 asymmetric unit, as calculated using MolProbity, a structure validation web server that evaluates atomic model quality (Chen and colleagues, 2010, PMID: 20057044). *Calculated in UCSF Chimera (Pettersen and colleagues, 2004, PMID: 15264254). The inhibitor had no clashes and was given MolProbity score 1.65 (91st percentile). CVB, Coxsackievirus B.
(DOCX)

S3 Table PISA analysis of the binding pocket surface area, and conservation of the pocket across CVB3 and enterovirus B group. The PISA server (Krissinel and Henrick, 2007, PMID: 17681537) was used to identify interfacing residues to the drug within the interprotomer binding pocket. These are listed here, along with the residue characteristics as calculated by PISA. Conservation of the pocket is shown with residues of different identity (after alignment) ordered by occurrence from the polyprotein sequences listed below, for sequenced CVB3 and enterovirus B comparators. Similarity scores are calculated using the average Grantham distance, indicating physicochemical differences between residues. CVB, Coxsackievirus B.; PISA, Proteins, Interfaces, Structures, and Assemblies.
(DOCX)

S4 Table RMSD evaluation of the pocket: Existing atomic models of mature picornaviruses were matched to our calculated model VP1 residues 74–80, 97–98, 155–160, 230–235, and VP3 residues 232–236. RMSD for these residues were calculated for atoms Cα and Cβ (to give an indication to side chain orientation) using UCSF Chimera (Pettersen and colleagues, 2004, PMID: 15264254). We observed that when the Cα distance was small (between 0.6 and 0.8 Å), this correlated with the highest antiviral activity. RMSD, root mean square deviation; UCSF, University California San Francisco.
(DOCX)

S1 raw data (XLSX)

## Abbreviations

A-particle: altered particle
CAR: Coxsackievirus and adenovirus receptor
COPD: chronic obstructive pulmonary disease
CPE: cytopathic effect
CVA: Coxsackievirus A
CVB: Coxsackievirus B
cVDPV: circulating vaccine-derived poliovirus
DAF: decay-accelerating factor
E-1: echovirus 1
E-7: echovirus 7
EC_50_: 50% effective concentration
EM: electron microscopy
EV: enterovirus
EVA: enterovirus A
EVB: enterovirus B
EVC: enterovirus C
EVD: enterovirus D
EVD68: enterovirus D68
EVA71: enterovirus A71
FBS: fetal bovine serum
hCAR: human Coxsackie virus and adenovirus receptor
MDFF: molecular dynamics flexible fitting
MOI: multiplicity of infection
MTS/PMS: 3-(4,5-dimethylthiazol-2-yl)-5-(3-carboxymethoxyphenyll-2-(4-sulfophenyl)-2-H-tetrazolium, inner salt)/phenazine methosulfate
PDB: Protein Data Bank
PISA: Proteins, Interfaces, Structures, and Assemblies
PV: poliovirus
PV1: poliovirus type I
qRT-PCR: quantitative reverse transcription PCR
RMSD: root mean square deviation
RV: rhinovirus
RVA: rhinovirus A
RVB: rhinovirus B
SAR: structure-activity relationship
SMILES: simplified molecular-input line-entry system
TCID_50_: 50% tissue culture infective dose
UCSF: University California San Francisco
