# Supplementary figures and images for: A novel druggable interprotomer pocket in the capsid of rhino- and enteroviruses
Source: PLoS Biol. 2019 Jun 11;17(6):e3000281. doi: 10.1371/journal.pbio.3000281 (PMC6559632; doi:10.1371/journal.pbio.3000281)

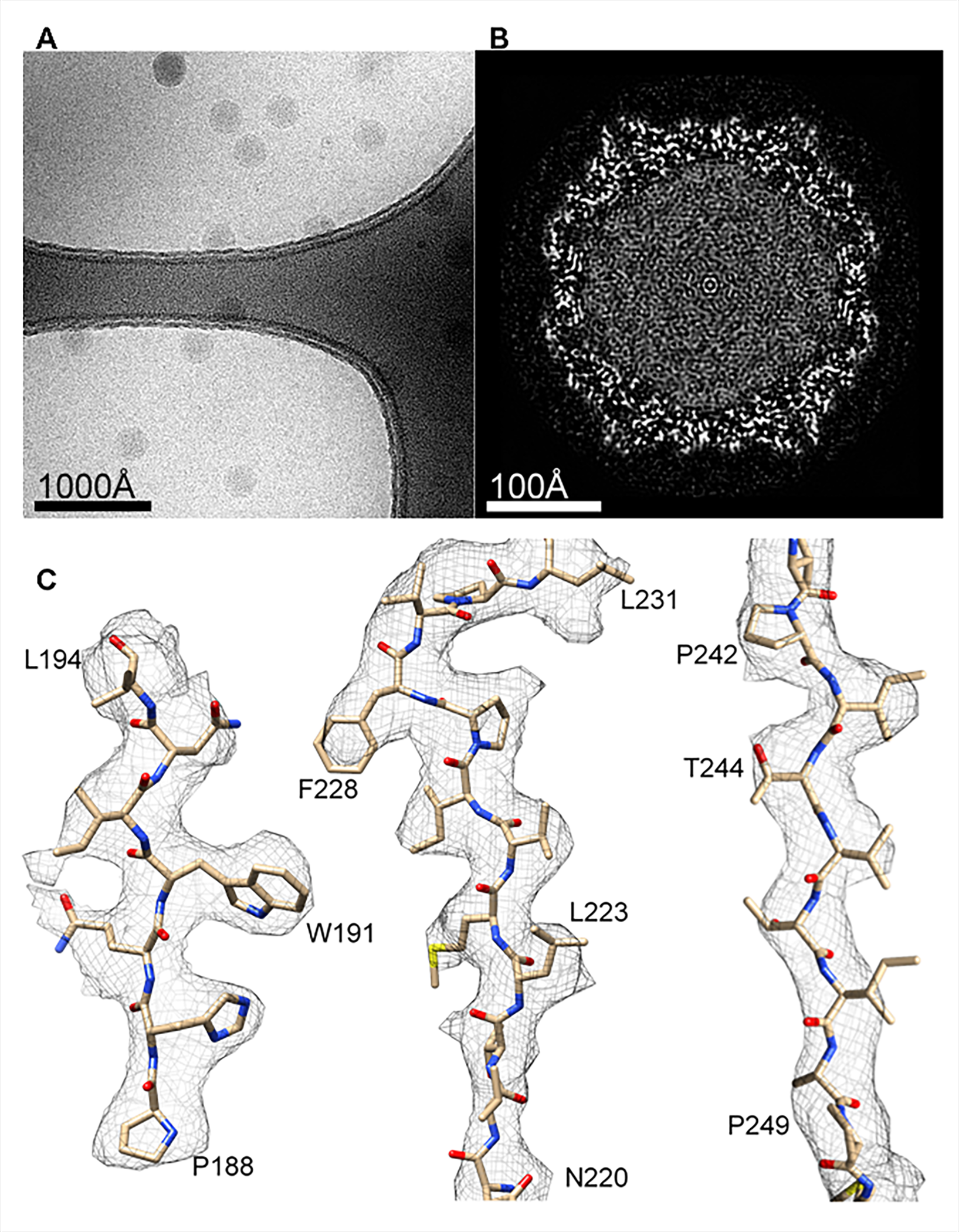

Supplement: S1 Fig — (A) An example micrograph from data collection processed for this paper. (B) Grayscale central section of the reconstruction. (C) Representative electron density and model fit for selected residues in VP2. (TIF) [file pbio.3000281.s001.tif]

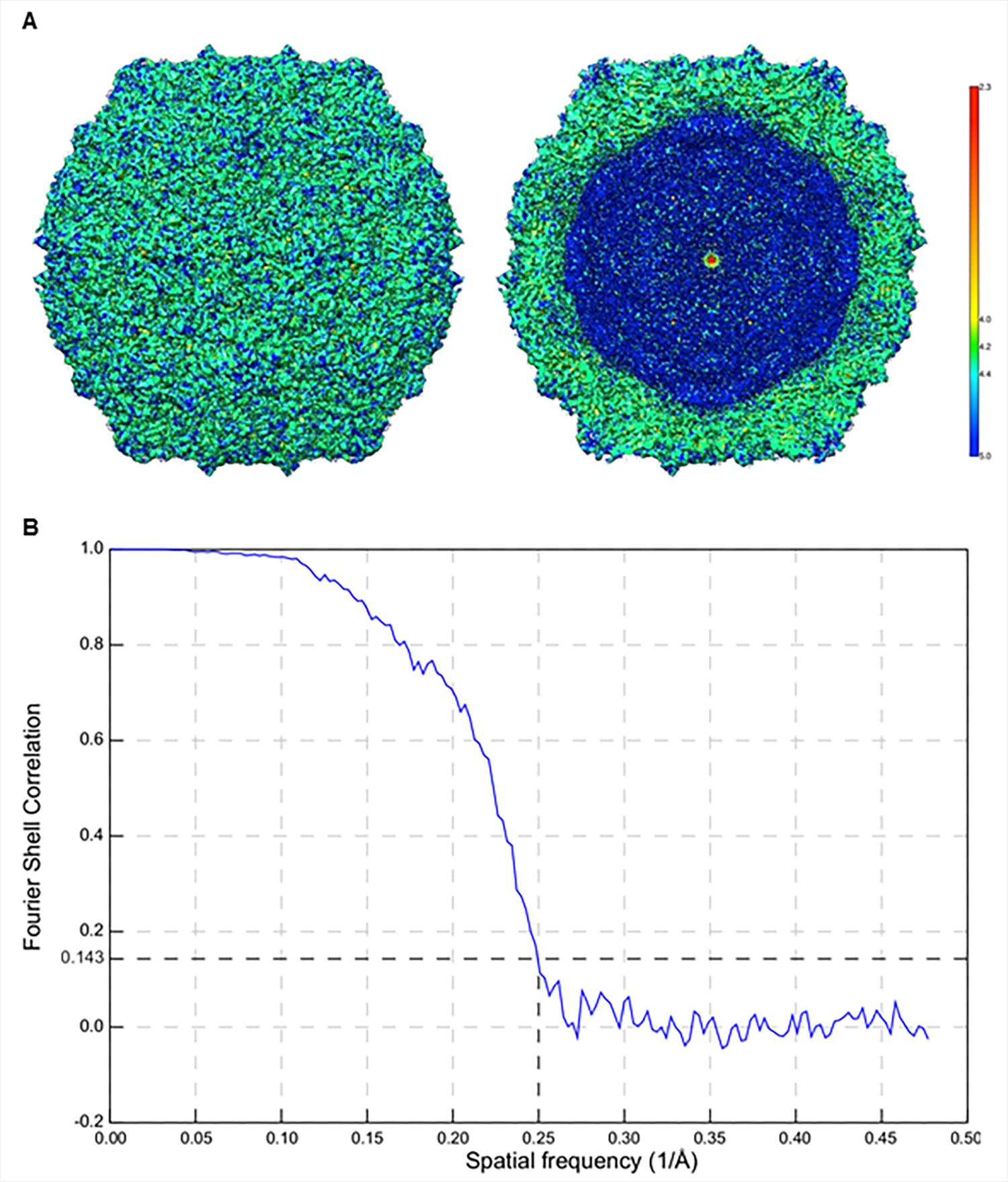

Supplement: S2 Fig — (A) Estimation of local resolution of reconstruction using ResMap (Kucukelbir and colleagues, 2014, PMID: 24213166), showing the capsid protein at approximately 4 Å on unsharpened full map and central section. (B) Fourier shell correlation calculated in Relion, with a resolution estimate of 4.0 Å as assessed at the 0.143 criterion. (TIF) [file pbio.3000281.s002.tif]

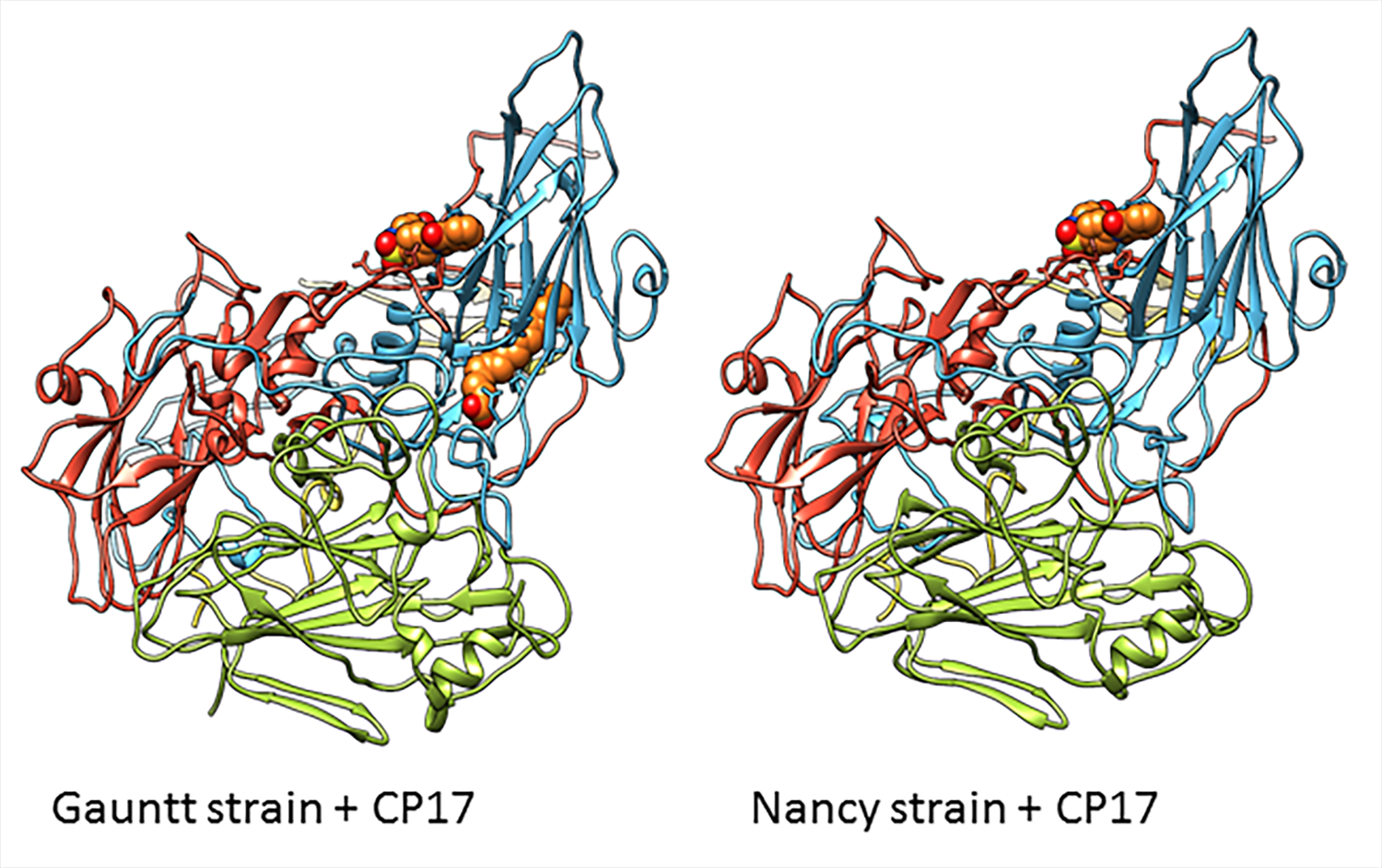

Supplement: S3 Fig — The hydrophobic pleconaril drug-binding pocket of the VP1 β-sandwich is shown in the Gauntt strain (left) with the pocket factor present (PDB ID: 3jd7), which is missing from the solved Nancy strain (right). Pleconaril enters this hydrophobic pocket and displaces the pocket factor. CP17, in contrast, targets a region on the outside of the VP1 β-sandwich (modeled into both strains). CP17, compound 17. (TIF) [file pbio.3000281.s003.tif]

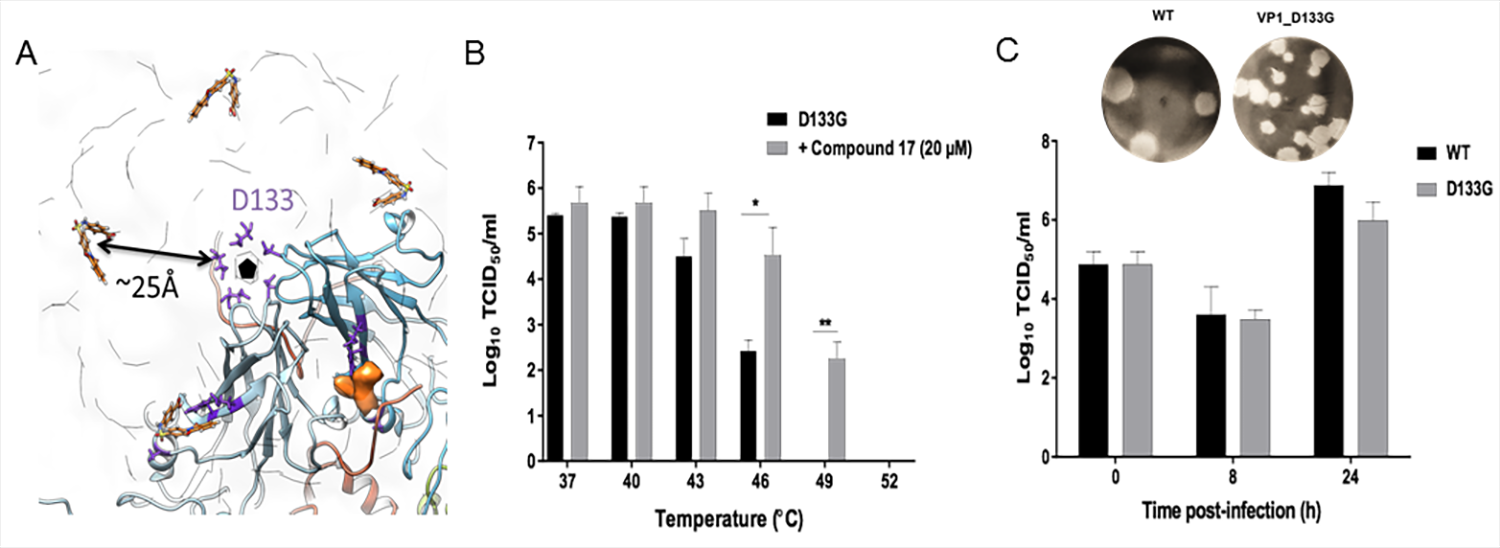

Supplement: S4 Fig — (A) Map of the VP1 residues involved in compound 17 resistance: residues F76, E78, and A98 map to the binding pocket identified using cryo-EM, and D133 is located in the central ion channel at the 5-fold vertex regions. (B) Thermostability assay: a high-titered stock of CVB3 VP1_D133G variant was incubated at different temperatures in the presence or absence of 20 μM compound 17. The residual infectivity of the virus was determined by end-point titration. Values are the mean ± SD of three independent experiments. Statistical differences (*p < 0.05, **p < 0.01) were analyzed by the unpaired t test. (C) Growth kinetics and plaque phenotyping of VP1_D133G variant: the infectious virus titer of CVB3 WT and VP1_D133G variant at different time points was determined by end-point titration. The plaque phenotype was determined by infecting Vero A cells with 10-fold serial dilution of each virus stock followed by addition of an agarose overlay. On day 3 postinfection, the viral plaques were visualized by Giemsa staining. The raw data of figures are presented in S1 raw data. CVB, Coxsackievirus B; WT, wild-type. (TIF) [file pbio.3000281.s004.tif]

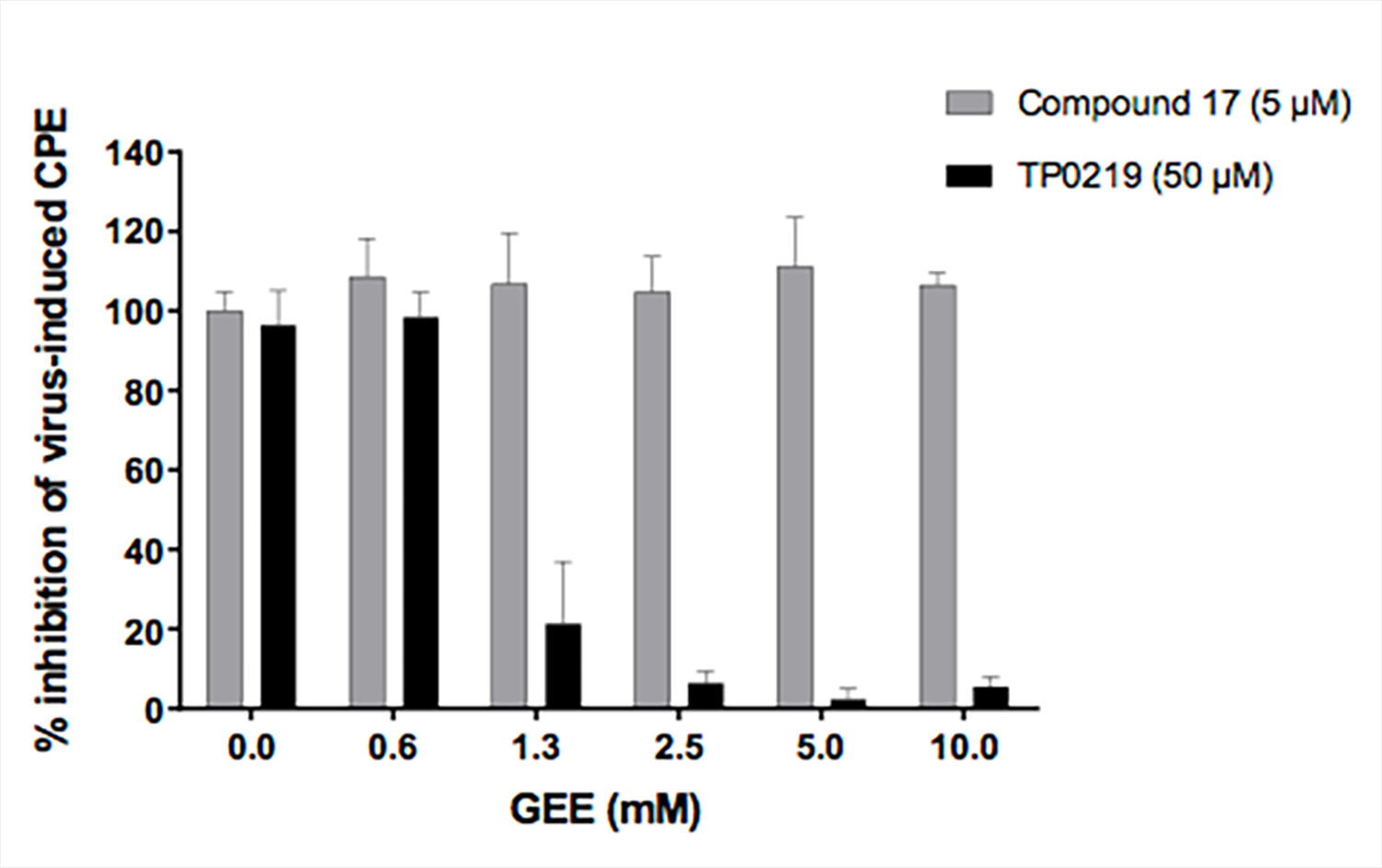

Supplement: S5 Fig — Effect of GEE on the antiviral activity of compound 17. Vero A cells were treated with 2-fold serial dilutions of the GEE (highest concentration 10 mM). Following 1 h of incubation, a fixed concentration of compound 17 (5 μM) or TP0219 (50 μM) was added to the GEE-treated and non-treated cells, followed by infection with CVB3 WT at an MOI of 0.01. On day 3 postinfection, the effect of GEE treatment on the antiviral activity of the tested compounds was quantified using the MTS/PMS method. Data represented are percentages of untreated controls and are mean values ± SD of three independent experiments. The raw data of figures are presented in S1 raw data. CVB, Coxsackievirus B; GEE, glutathione ethyl ester; MOI, multiplicity of infection; MTS/PMS, 3-(4,5-dimethylthiazol-2-yl)-5-(3-carboxymethoxyphenyll-2-(4-sulfophenyl)-2-H-tetrazolium; WT, wild-type. (TIF) [file pbio.3000281.s005.tif]

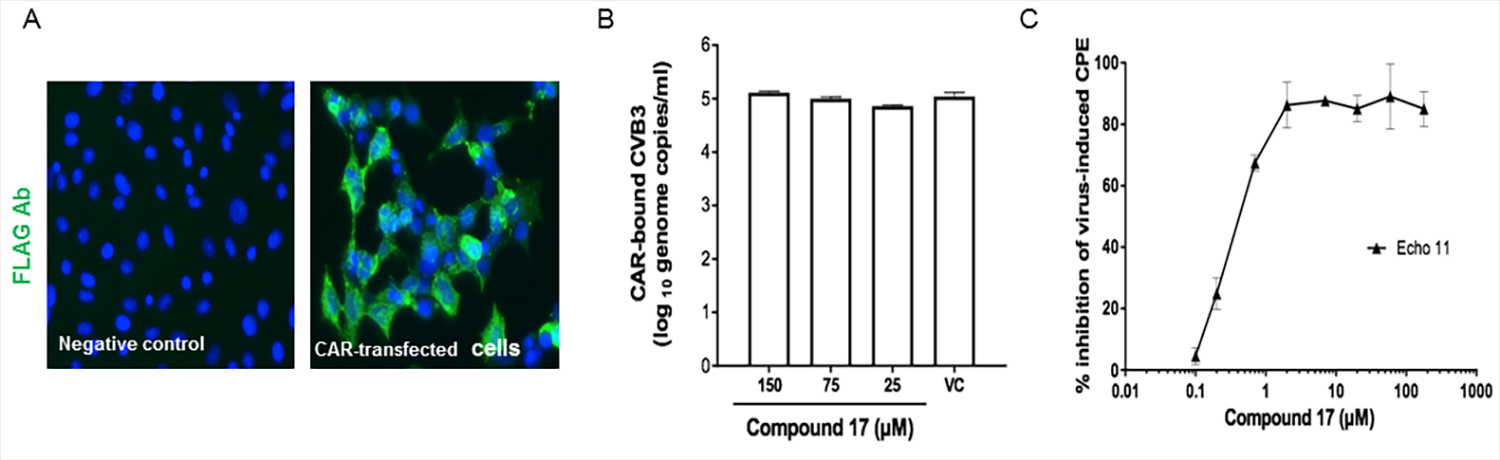

Supplement: S6 Fig — (A) Immunofluorescence image for expression of C-terminal flag-tagged CAR in HEK239T cells. (B) Immunoprecipitation of CVB3 with flag-tagged CAR in presence or absence of compound 17 as quantified by qRT-PCR. (C) In vitro antiviral activity of compound 17 against E-11 (a DAF-dependent enterovirus B) in a CPE reduction assay. The raw data of figures are presented in S1 raw data. CAR, Coxsackievirus and adenovirus receptor; CPE, cytopathic effect; CVB, Coxsackievirus B; DAF, decay-accelerating factor; E-11, echovirus 11; qRT-PCR, quantitative reverse transcription PCR. (TIF) [file pbio.3000281.s006.tif]

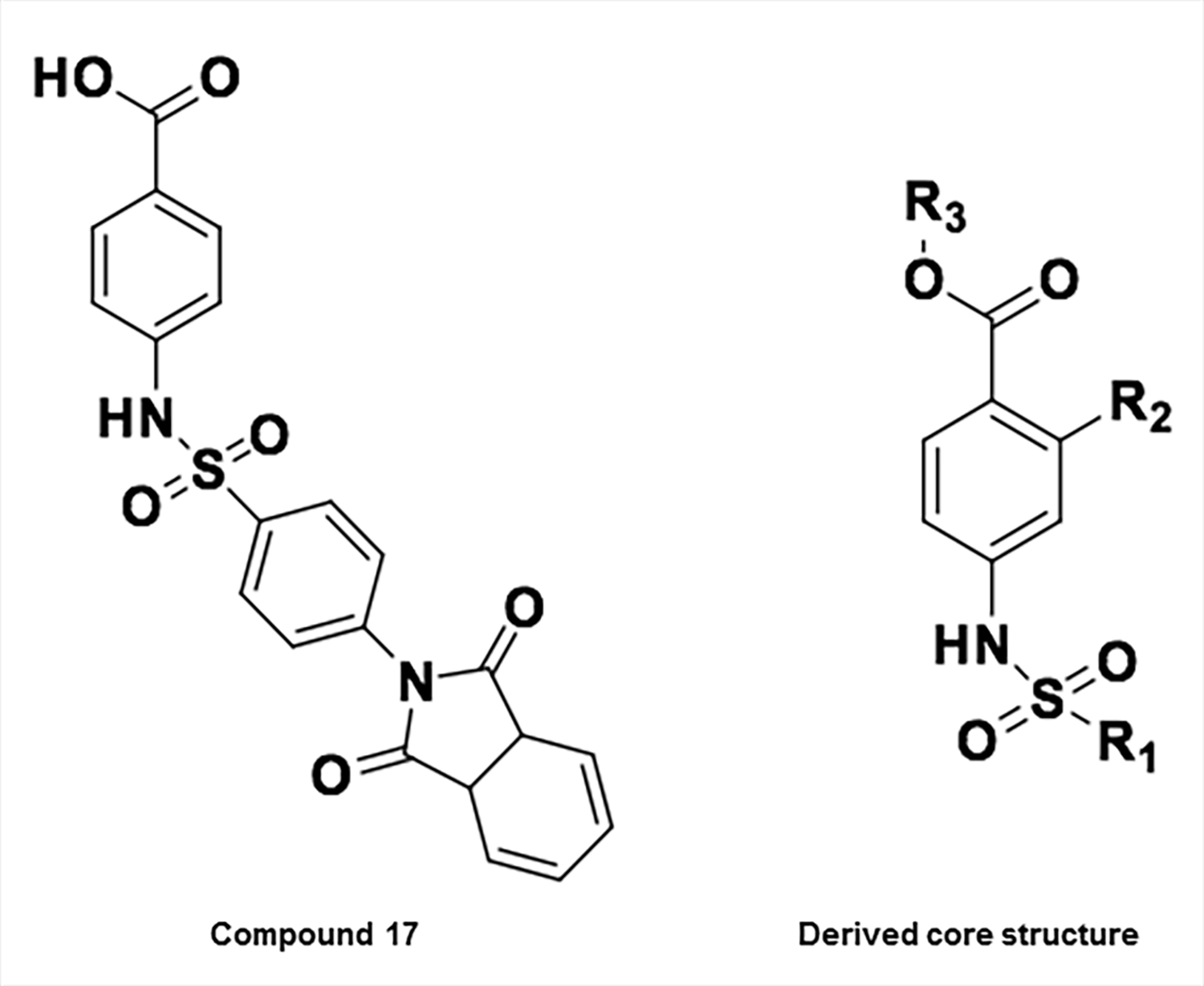

Supplement: S7 Fig — (TIF) [file pbio.3000281.s007.tif]

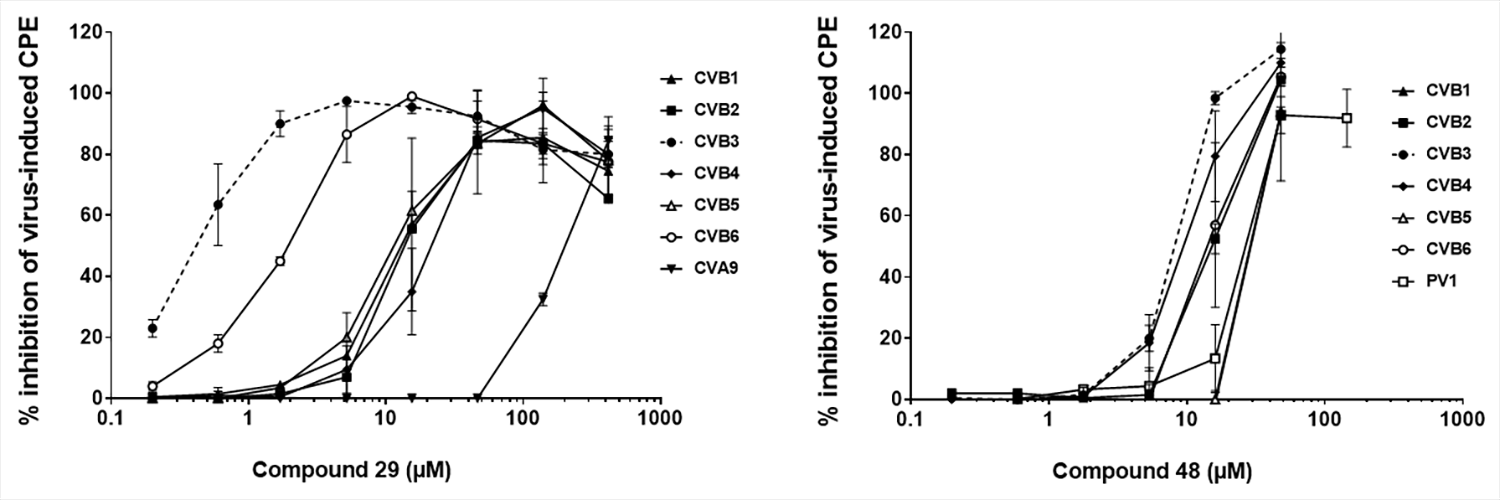

Supplement: S8 Fig — Dose-response antiviral activity of (A) compound 29 and (B) compound 48 on the replication of selected enteroviruses in a CPE reduction assay. Data are mean values ± SD of at least two independent experiments. The raw data of figures are presented in S1 raw data. CPE, cytopathic effect. (TIF) [file pbio.3000281.s008.tif]
