# Supplementary material for: A novel druggable interprotomer pocket in the capsid of rhino- and enteroviruses
Source: PLoS Biol. 2019 Jun 11;17(6):e3000281. doi: 10.1371/journal.pbio.3000281 (PMC6559632; doi:10.1371/journal.pbio.3000281)
Supplement: S1 Table — (DOCX) [file pbio.3000281.s009.docx]

| **Compound** | | | | **EC_50_ ± SD/µM** | | | | | | | | | | **CC_50_ ± SD/µM** | | |
| --- | --- | --- | --- | --- | --- | --- | --- | --- | --- | --- | --- | --- | --- | --- | --- | --- |
| **ID** | **R_1_** | **R_2_** | **R_3_** | **CVB1** | **CVB2** | **CVB3** | **CVB4** | **CVB5** | **CVB6** | **CVA21** | **EVD68** | **PV1** | **RVB14** | **BGM** | **Hela** | **Vero A** |
| 17 |  | -H | -H | 3.1±0.8 | >296 | 0.7±0.1 | 16±0.3 | 37±5 | 9±0.5 | >296 | >296 | >400 | >296 | >400 | >296 | >296 |
| 20 | 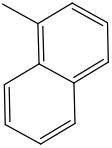 | -OH | -H | NA | 82±5 | 41±2 | 95±1 | NA | 12±1 | NA | NA | 255±21 | NA | >470 | 301±16 | >470 |
| 21 | 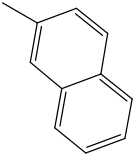 | -OH | -H | NA | NA | 6.4±1.2 | 120±3 | NA | 23±3 | NA | NA | 275±18 | NA | >470 | 306±5 | >470 |
| 22 | 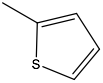 | -OH | -H | NA | NA | 1.3±0.0 | 338±100 | NA | 25±3 | NA | NA | NA | NA | >550 | >550 | >550 |

| **Compound** | | | | **EC_50_ ± SD/µM** | | | | | | | | | | **CC_50_ ± SD/µM** | | |
| --- | --- | --- | --- | --- | --- | --- | --- | --- | --- | --- | --- | --- | --- | --- | --- | --- |
| **ID** | **R_1_** | **R_2_** | **R_3_** | **CVB1** | **CVB2** | **CVB3** | **CVB4** | **CVB5** | **CVB6** | **CVA21** | **EVD68** | **PV1** | **RVB14** | **BGM** | **Hela** | **Vero A** |

| 23 | 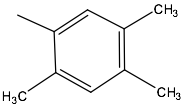 | -OH | -H | 174±56 | 191±  22 | 21±8 | 126±26 | NA | 35±0 | NA | NA | 109±37 | NA | >480 | 116±14 | >480 |
| --- | --- | --- | --- | --- | --- | --- | --- | --- | --- | --- | --- | --- | --- | --- | --- | --- |
| 24 |  | -OH | -H | 7.4±4.6 | NA | 0.6±0.1 | 61±15 | NA | 12±0 | 38±3 | NA | 277±72 | NA | >480 | >480 | >480 |
| 25 |  | -OH | -H | 461±51 | NA | 0.3±0.1 | 155±62 | NA | 20±4 | NA | NA | 316±2 | 119±13 | >480 | 316±4 | >480 |
| 26 | 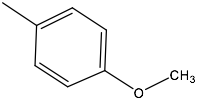 | -OH | -H | NA | NA | 0.9±0.3 | NA | NA | 37±1 | NA | NA | NA | NA | >500 | >500 | >500 |
| 27 | 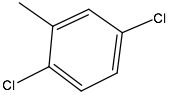 | -OH | -H | NA | 357±  10 | 21±1 | 214±1 | NA | 6.5±3.3 | NA | NA | 337±5 | NA | >450 | 288±6 | >450 |
| 28 | 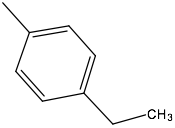 | -OH | -H | NA | 346±  16 | 6.2±3.1 | 286±22 | NA | 34±2 | NA | NA | 338±9 | NA | >500 | 331±1 | >500 |
| 29 |  | -OH | -H | 16±10 | 14±0 | 0.4±0.1 | 21±5 | 12±2 | 2.0±0.0 | 203±17 | NA | 307±30 | NA | >400 | >400 | >400 |
| 30 | 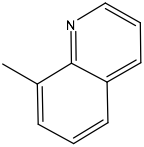 | -OH | -H | NA | NA | 71±31 | 281±9 | NA | 15±2 | NA | 79±4 | NA | 25±2 | >450 | >450 | >450 |

| **Compound** | | | | **EC_50_ ± SD/µM** | | | | | | | | | | **CC_50_ ± SD/µM** | | |
| --- | --- | --- | --- | --- | --- | --- | --- | --- | --- | --- | --- | --- | --- | --- | --- | --- |
| **ID** | **R_1_** | **R_2_** | **R_3_** | **CVB1** | **CVB2** | **CVB3** | **CVB4** | **CVB5** | **CVB6** | **CVA21** | **EVD68** | **PV1** | **RVB14** | **BGM** | **Hela** | **Vero A** |

| 31 | 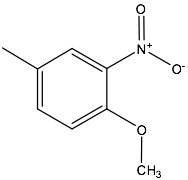 | -OH | -H | NA | NA | 2.9±0.4 | 198±55 | NA | NA | NA | NA | NA | NA | >450 | >450 | >450 |
| --- | --- | --- | --- | --- | --- | --- | --- | --- | --- | --- | --- | --- | --- | --- | --- | --- |
| 32 | 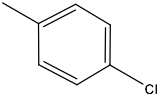 | -OH | -H | NA | NA | 3.1±0.3 | 290±23 | NA | 37±4 | NA | NA | NA | NA | >500 | >500 | >500 |
| 33 | 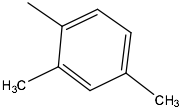 | -OH | -H | NA | 305±5 | 24±8 | 256±23 | NA | 14±2 | NA | NA | NA | NA | >500 | 314±11 | >500 |
| 34 | 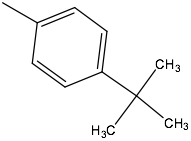 | -OH | -H | NA | 99±4 | 9.4±1.2 | 65±22 | NA | 81±6 | NA | NA | 115±11 | 36±2 | 306±4 | 105±2 | 237±10 |
| 35 | 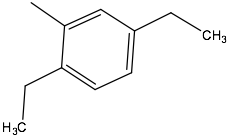 | -OH | -H | NA | 148±2 | 22±5 | 96±8 | NA | 9.0±0.2 | NA | NA | NA | NA | 151±18 | 32±1 | 304±17 |
| 36 | 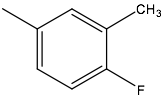 | -OH | -H | NA | NA | 1.7±0.5 | 281±21 | NA | 35±1 | NA | NA | NA | NA | >500 | >500 | >500 |
| 37 | 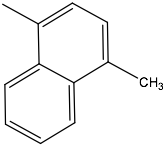 | -OH | -H | 247±40 | 51±6 | 34±4 | 65±12 | 310±43 | 18±3 | NA | NA | 301±1 | 47±2 | >450 | 98±1 | >450 |
| 38 | 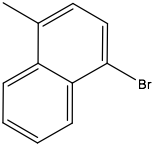 | -OH | -H | NA | 20±0 | 22±1 | 25±6 | NA | 19±5 | NA | NA | 78±3 | NA | 165±6 | 74±3 | 57±1 |

| **Compound** | | | | **EC_50_ ± SD/µM** | | | | | | | | | | **CC_50_ ± SD/µM** | | |
| --- | --- | --- | --- | --- | --- | --- | --- | --- | --- | --- | --- | --- | --- | --- | --- | --- |
| **ID** | **R_1_** | **R_2_** | **R_3_** | **CVB1** | **CVB2** | **CVB3** | **CVB4** | **CVB5** | **CVB6** | **CVA21** | **EVD68** | **PV1** | **RVB14** | **BGM** | **Hela** | **Vero A** |

| 39 | 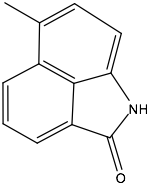 | -H | -H | NA | NA | 155±156 | NA | NA | NA | NA | NA | NA | NA | >400 | >400 | >400 |
| --- | --- | --- | --- | --- | --- | --- | --- | --- | --- | --- | --- | --- | --- | --- | --- | --- |
| 40 | 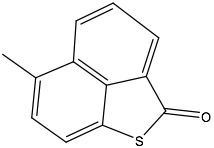 | -H | -H | NA | NA | 237±46 | 319±72 | NA | NA | NA | NA | 250±0 | NA | >400 | >400 | >400 |
| 41 | 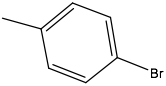 | -OH | -H | NA | NA | 4.6±3.0 | 232±14 | NA | 36±5 | NA | NA | 303±11 | NA | >400 | 289±5 | >400 |
| 42 | 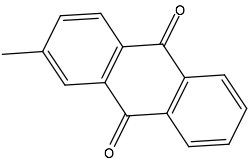 | -H | -H | NA | NA | 72±60 | 114±9 | NA | NA | NA | NA | NA | NA | 132±47 | 23±1 | 199±18 |
| 43 | 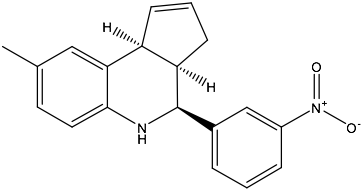 | -H | -H | 32±10 | NA | 10±5 | 60±6 | NA | 59±5 | NA | NA | 180±45 | NA | 279±1 | 23±1 | 234±2 |
| 44 | 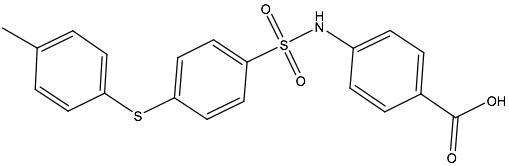 | -H | -H | 1.5±0.7 | NA | 0.4±0.1 | 150±0 | NA | 6.4±0.4 | NA | NA | NA | NA | >250 | >250 | >250 |
| 45 | 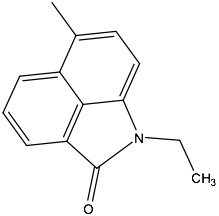 | -H | -H | NA | NA | 189±26 | 80±11 | NA | NA | NA | NA | NA | NA | >400 | 265±12 | >400 |
| 46 | 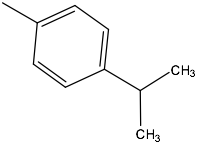 | -H | -H | 112±6 | NA | 5.6±1.8 | 74±7 | 192±70 | 66±21 | NA | NA | 122±16 | 130±19 | >500 | 338±12 | >500 |

| **Compound** | | | | **EC_50_ ± SD/µM** | | | | | | | | | | **CC_50_ ± SD/µM** | | |
| --- | --- | --- | --- | --- | --- | --- | --- | --- | --- | --- | --- | --- | --- | --- | --- | --- |
| **ID** | **R_1_** | **R_2_** | **R_3_** | **CVB1** | **CVB2** | **CVB3** | **CVB4** | **CVB5** | **CVB6** | **CVA21** | **EVD68** | **PV1** | **RVB14** | **BGM** | **Hela** | **Vero A** |

| 47 | 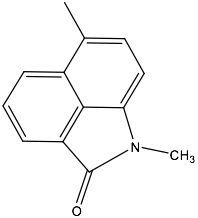 | -H | -H | NA | NA | 210±91 | 306±42 | NA | NA | NA | NA | NA | NA | >400 | 271±10 | >400 |
| --- | --- | --- | --- | --- | --- | --- | --- | --- | --- | --- | --- | --- | --- | --- | --- | --- |
| 48 |  | -H | -H | 29±0.4 | 15±2 | 8.2±0.2 | 8.6±0.8 | 29±5 | 15±6 | NA | NA | 27±1 | NA | 173±35 | 64±44 | 197±7 |
| 49 | 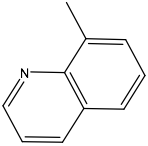 | -H | -H | NA | NA | 39±10 | 35±2 | NA | 60±1 | NA | 79±8 | ND | 33±1 | ND | >300 | >300 |
| 50 | 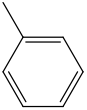 | -H | -H | NA | NA | 3.8±0.8 | NA | NA | 58±12 | NA | NA | ND | NA | ND | >350 | >350 |
| 51 | 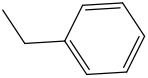 | -H | -H | NA | NA | NA | NA | NA | NA | NA | NA | ND | NA | ND | >340 | >340 |
| 52 | 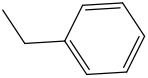 | 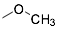 | -H | NA | NA | NA | NA | NA | NA | NA | NA | ND | NA | ND | >300 | >300 |
| 53 |  | -H | -H | 85±6 | 4±0.3 | 11±3 | 9.4±1.3 | NA | 1±0.1 | NA | NA | ND | 13±1 | ND | 107±27 | >300 |
| 54 |  | 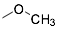 | H | NA | NA | NA | NA | NA | 16±1 | NA | NA | ND | NA | ND | 137±37 | >250 |

| **Compound** | | | | **EC_50_ ± SD/µM** | | | | | | | | | | **CC_50_ ± SD/µM** | | |
| --- | --- | --- | --- | --- | --- | --- | --- | --- | --- | --- | --- | --- | --- | --- | --- | --- |
| **ID** | **R_1_** | **R_2_** | **R_3_** | **CVB1** | **CVB2** | **CVB3** | **CVB4** | **CVB5** | **CVB6** | **CVA21** | **EVD68** | **PV1** | **RVB14** | **BGM** | **Hela** | **Vero A** |

| 55 | 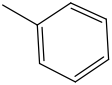 | -OH | -CH_3_ | NA | NA | NA | NA | NA | NA | NA | NA | ND | NA | ND | 118±37 | >300 |
| --- | --- | --- | --- | --- | --- | --- | --- | --- | --- | --- | --- | --- | --- | --- | --- | --- |
| 56 | 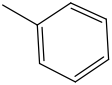 | -OH | -H | NA | NA | 0.7±0.1 | NA | NA | 4.8±0.2 | NA | NA | ND | NA | ND | >330 | >330 |
| 57 | 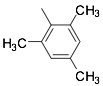 | -OH | -CH_3_ | NA | NA | NA | NA | NA | NA | NA | NA | ND | NA | ND | 11±0 | 28±2 |
| 58 | 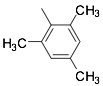 | -OH | -H | NA | NA | 8.5±2.5 | NA | NA | 27±5 | NA | NA | ND | NA | ND | 89±0.4 | 294±1 |
| 59 |  | -OH | -CH_3_ | NA | NA | NA | NA | NA | NA | NA | NA | ND | NA | ND | 11±4 | 16.5±1 |
| 60 |  | -OH | -H | 67±9 | 8.4±1.1 | 12±3 | 12±2 | NA | 0.2±0 | NA | NA | ND | NA | ND | 75±26 | >250 |
| 61 | 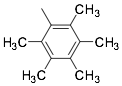 | -OH | -CH_3_ | NA | NA | NA | NA | NA | NA | NA | NA | ND | NA | ND | 8±2 | 22±6 |
| 62 | 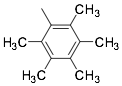 | -OH | -H | 50±3 | 35±3 | 2.8±0.8 | NA | NA | 32±1 | NA | NA | ND | NA | ND | 49±6 | 250±6 |
| 63 | -CH_3_-CH_3_ | -OH | -H | 35±5 | NA | 0.9±0.2 | 6.1±1 | NA | 22±3 | NA | NA | ND | NA | ND | >400 | >400 |

| **Compound** | | | | **EC_50_ ± SD/µM** | | | | | | | | | | **CC_50_ ± SD/µM** | | |
| --- | --- | --- | --- | --- | --- | --- | --- | --- | --- | --- | --- | --- | --- | --- | --- | --- |
| **ID** | **R_1_** | **R_2_** | **R_3_** | **CVB1** | **CVB2** | **CVB3** | **CVB4** | **CVB5** | **CVB6** | **CVA21** | **EVD68** | **PV1** | **RVB14** | **BGM** | **Hela** | **Vero A** |

| 64 |  | -F | -H | NA | NA | 36±7 | NA | NA | 3.8±0.5 | NA | 8.7±0.6 | ND | NA | ND | 63±1 | >250 |
| --- | --- | --- | --- | --- | --- | --- | --- | --- | --- | --- | --- | --- | --- | --- | --- | --- |
| 65 | 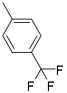 | -OH | -CH_3_ | NA | NA | NA | NA | NA | NA | NA | NA | ND | NA | ND | 15±1 | 20±6 |
| 66 | 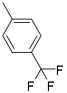 | -OH | -H | NA | NA | 2.4±1.5 | NA | NA | 67±8 | NA | NA | ND | NA | ND | >250 | >250 |
| 67 | 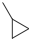 | -OH | -CH_3_ | 330±7 | 363±0.5 | 63±11 | 125±38 | NA | NA | NA | NA | ND | NA | ND | >350 | >350 |
| 68 | 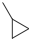 | -OH | -H | NA | NA | 4.7±0.4 | NA | NA | NA | NA | NA | ND | NA | ND | >350 | >350 |
| 69 | 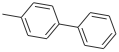 | -OH | -CH_3_ | NA | NA | NA | NA | NA | NA | NA | NA | ND | NA | ND | 9±1 | 16±5 |
| 70 | 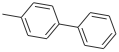 | -OH | -H | NA | NA | 3±1.3 | NA | NA | 15±2 | NA | NA | ND | NA | ND | 58±24 | 254±4 |
| 71 | 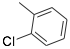 | -OH | -CH_3_ | NA | NA | 20±8 | 21±6 | NA | NA | NA | NA | ND | NA | ND | 13±7 | 62±7 |
| 72 | 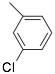 | -OH | -H | NA | NA | 3.2±1.5 | 116±4 | NA | 3.1±0.3 | NA | NA | ND | NA | ND | 177±57 | >300 |
| 73 | 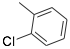 | -OH | -CH_3_ | NA | NA | NA | 28±7 | NA | 13±0.7 | NA | NA | ND | NA | ND | 71±53 | 110±4 |

| **Compound** | | | | **EC_50_ ± SD/µM** | | | | | | | | | | **CC_50_ ± SD/µM** | | |
| --- | --- | --- | --- | --- | --- | --- | --- | --- | --- | --- | --- | --- | --- | --- | --- | --- |
| **ID** | **R_1_** | **R_2_** | **R_3_** | **CVB1** | **CVB2** | **CVB3** | **CVB4** | **CVB5** | **CVB6** | **CVA21** | **EVD68** | **PV1** | **RVB14** | **BGM** | **Hela** | **Vero A** |

| 74 | 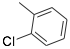 | -OH | -H | NA | NA | 13±0.3 | NA | NA | 6.6±0.2 | NA | NA | ND | NA | ND | 168±24 | >300 |
| --- | --- | --- | --- | --- | --- | --- | --- | --- | --- | --- | --- | --- | --- | --- | --- | --- |
| 75 | 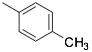 | -OH | -CH_3_ | NA | NA | 15±1.1 | NA | NA | NA | NA | NA | ND | NA | ND | 24±17 | 112±3 |
| 76 | 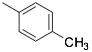 | -OH | -H | NA | NA | 1.1±0.4 | NA | NA | 15±1 | NA | NA | ND | NA | ND | >300 | >300 |
| 77 |  | -CH_3_ | -H | NA | 98±3 | 11±4 | 59±5 | NA | 18±1 | NA | NA | ND | 45±8 | ND | 174±0 | >250 |
| 78 | 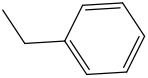 | -OH | -H | NA | NA | 99±67 | NA | NA | NA | NA | NA | ND | NA | ND | >300 | >300 |
| 79 | 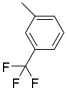 | -OH | -CH_3_ | NA | NA | NA | NA | NA | NA | NA | NA | ND | NA | ND | 6±1 | 15±2 |
| 80 | 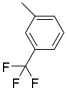 | -OH | -H | NA | NA | 7.2±3.9 | 116±24 | NA | NA | NA | NA | ND | NA | ND | 208±59 | >250 |
| 81 | 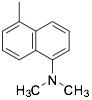 | -OH | -CH_3_ | NA | NA | NA | NA | NA | NA | NA | NA | ND | NA | ND | 6±3 | 16±1 |

EC_50_ values are mean values +/- standard deviation from at least two independent experiments. EC_50_ was calculated in a CPE-reduction assay with MTS read-out.

CC_50_ values are mean values +/- standard deviation from at least two independent experiments. NA, Not active, ND, Not determined
