# Supplementary material for: A novel druggable interprotomer pocket in the capsid of rhino- and enteroviruses
Source: PLoS Biol. 2019 Jun 11;17(6):e3000281. doi: 10.1371/journal.pbio.3000281 (PMC6559632; doi:10.1371/journal.pbio.3000281)
Supplement: S2 Table — *Calculated in UCSF Chimera (Pettersen and colleagues, 2004, PMID: 15264254). The inhibitor had no clashes and was given MolProbity score 1.65 (91st percentile). CVB, Coxsackievirus B. (DOCX) [file pbio.3000281.s010.docx]

| **Model** | **Residues** |  |
| --- | --- | --- |
| VP1 | 13-281 |  |
| VP2 | 8-262 |  |
| VP3 | 1-238 |  |
| VP4 | 2-12,25-69 |  |
|  |  |  |
| **MolProbity statistics** | **Score** |  |
| Clashscore, all atoms | 0 | 100^th^ percentile |
| Poor rotamers | 9 | 1.3% |
| Favored rotamers | 651 | 92% |
| Ramachandran outliers | 30 | 3.7% |
| Ramachandran favored | 698 | 86% |
| MolProbity score | 1.21 | 99^th^ percentile |
| Cβ deviations >0.25 Å | 2 | 0.3% |
| Bad bonds | 0 / 6537 | 0.0% |
| Bad angles | 41 / 8923 | 0.5% |
| Cis Prolines | 0 / 54 | 0.0% |
| Cis nonProlines | 0 / 759 | 0.0% |
| Twisted Peptides | 0 / 813 | 0.0% |
| RMSD with 1COV * | 0.89 Å |  |
