# Supplementary material for: A novel druggable interprotomer pocket in the capsid of rhino- and enteroviruses
Source: PLoS Biol. 2019 Jun 11;17(6):e3000281. doi: 10.1371/journal.pbio.3000281 (PMC6559632; doi:10.1371/journal.pbio.3000281)
Supplement: S3 Table — The PISA server (Krissinel and Henrick, 2007, PMID: 17681537) was used to identify interfacing residues to the drug within the interprotomer binding pocket. These are listed here, along with the residue characteristics as calculated by PISA. Conservation of the pocket is shown with residues of different identity (after alignment) ordered by occurrence from the polyprotein sequences listed below, for sequenced CVB3 and enterovirus B comparators. Similarity scores are calculated using the average Grantham distance, indicating physicochemical differences between residues. CVB, Coxsackievirus B.; PISA, Proteins, Interfaces, Structures, and Assemblies. (DOCX) [file pbio.3000281.s011.docx]

| **Residue** | **Surface area** | **Buried surface** | **Solvation energy** | **CVB3 identity** | **CVB3 Grantham** | **EVB identity** | **EVB Grantham** |
| --- | --- | --- | --- | --- | --- | --- | --- |
| **VP1** |  |  |  |  |  |  |  |
| C73 | 36.36 | 10.58 | 0.23 | 100% | 100% | 100% | 100% |
| Y75 | 64.91 | 1.06 | 0.02 | 100% | 100% | 78% | 97% |
| F76 | 101.38 | 71.92 | 0.93 | 89% | 99% | 44% | 92% |
| T77 | 24.15 | 7.03 | 0.11 | 89% | 97% | 56% | 84% |
| E78 | 80.45 | 28.18 | -0.12 | 93% | 99% | 44% | 82% |
| D155 | 101.03 | 42.62 | 0.02 | 93% | 98% | 56% | 87% |
| S156 | 15.02 | 5.82 | 0 | 89% | 97% | 67% | 89% |
| Y157 | 166.2 | 24.66 | 0.29 | 95% | 99% | 100% | 100% |
| W159 | 15.07 | 7.23 | 0.04 | 100% | 100% | 100% | 100% |
| Q160 | 133.61 | 29.03 | 0.17 | 100% | 100% | 100% | 100% |
| R219 | 34.76 | 12.23 | -0.21 | 100% | 100% | 100% | 100% |
| R234 | 83.89 | 19.41 | -0.38 | 100% | 100% | 100% | 100% |
| **VP3** |  |  |  |  |  |  |  |
| Q233 | 110.06 | 26.8 | -0.08 | 100% | 100% | 100% | 100% |
| Q234 | 159.63 | 35.68 | -0.25 | 55% | 87% | 44% | 81% |
| N235 | 133.06 | 25.8 | 0.03 | 79% | 92% | 44% | 74% |
| F236 | 164.66 | 61.39 | 0.95 | 93% | 99% | 78% | 93% |

Genbank ID CVB3 strains:

AAA42931, AAA74400, AAB02228, AAB59927, AAG23918, AAG23919, AAG23920, AAT79531, AAV34211, AAV34212, AAX23955, AAX23956, AAX23957, AAX23958, AAX23959, AAX23960, AAX23961, AAX23962, ABW34429, ACH91034, ACJ05389, ACS34758, ACY40750, AEH42467, AEH42468, AFC88096, AFD33642, AFS18536, AFY09604, AGC54437, AGJ72750, AHK09954, AHM88285, AHY19027, AIZ97145, AIZ97146, AIZ97147, ALX38425, ALX38426, ALX38427, ALX38428, AMB56998, AMB56999, AMB57000, AMB57001, AMB57002, AMB57003, AMB57004, AMB57005, AMB57006, AMB57007, AMB57008, AMY98705, ARB48778, ARO77489

Genbank ID Enterovirus B strains:

CVB1: AAC00531; CVB2: AAD46138; CVB3: AEH42467; CVB4: AAL37156; CVB5: AAF21971; CVB6: AAF12719;
CVA9: AUF49637; E11: ABV00677
