# Supplementary material for: A novel druggable interprotomer pocket in the capsid of rhino- and enteroviruses
Source: PLoS Biol. 2019 Jun 11;17(6):e3000281. doi: 10.1371/journal.pbio.3000281 (PMC6559632; doi:10.1371/journal.pbio.3000281)
Supplement: S4 Table — RMSD for these residues were calculated for atoms Cα and Cβ (to give an indication to side chain orientation) using UCSF Chimera (Pettersen and colleagues, 2004, PMID: 15264254). We observed that when the Cα distance was small (between 0.6 and 0.8 Å), this correlated with the highest antiviral activity. RMSD, root mean square deviation; UCSF, University California San Francisco. (DOCX) [file pbio.3000281.s012.docx]

| **Species** | **Virus** | **wwPDB ID** | **Cα RMSD** | **Cβ RMSD** |
| --- | --- | --- | --- | --- |
| *Enterovirus B* | CVB3 | 1COV | 0.76 | 0.83 |
| *Enterovirus B* | E11 | 1H8T | 0.69 | 0.85* |
| *Enterovirus B* | CVA9 | 1D4M | 0.81 | 0.94 |
| *Enterovirus B* | E1 | 1EV1 | 0.92 | 1.05 |
| *Enterovirus B* | E7 | 2X5I | 0.79 | 0.82* |
| *Enterovirus D* | EV68 | 4WM8 | 1.31 | 1.56* |
| *Enterovirus C* | PV1 | 1HXS | 1.19^†^ | 1.51^†^ |
| *Enterovirus A* | CVA16 | 5C4W | 1.84 | 2.29 |
| *Enterovirus A* | EV71 | 3VBS | 1.82 | 2.20** |
| *Rhinovirus C* | RVC15 | 5K0U | 1.99 | 2.36** |

(*) A glycine residue was excluded from the Cβ RMSD calculation.
(**) Two glycine residues were excluded from the Cβ RMSD calculation.
(†) PV1 VP3 residue 236 is unmodeled in the deposited structure 1HXS and thus was excluded from analysis.
